# The rubber tree genome shows expansion of gene family associated with rubber biosynthesis

**DOI:** 10.1038/srep28594

**Published:** 2016-06-24

**Authors:** Nyok-Sean Lau, Yuko Makita, Mika Kawashima, Todd D. Taylor, Shinji Kondo, Ahmad Sofiman Othman, Alexander Chong Shu-Chien, Minami Matsui

**Affiliations:** 1 Centre for Chemical Biology, Universiti Sains Malaysia, 11900 Bayan Lepas, Penang, Malaysia; 2 Synthetic Genomics Research Group, RIKEN Center for Sustainable Resource Science, Biomass Engineering Research Division, Tsurumi, Yokohama, Kanagawa 230-0045, Japan; 3 Laboratory for Integrated Bioinformatics, RIKEN Center for Integrative Medical Sciences, Tsurumi, Yokohama, Kanagawa 230-0045, Japan; 4 Transdisciplinary Research Integration Center, National Institute of Polar Research, Tachikawa, Tokyo 190-8518, Japan; 5 School of Biological Sciences, Universiti Sains Malaysia, 11800 Minden, Penang, Malaysia

## Abstract

*Hevea brasiliensis* Muell. Arg, a member of the family Euphorbiaceae, is the sole natural resource exploited for commercial production of high-quality natural rubber. The properties of natural rubber latex are almost irreplaceable by synthetic counterparts for many industrial applications. A paucity of knowledge on the molecular mechanisms of rubber biosynthesis in high yield traits still persists. Here we report the comprehensive genome-wide analysis of the widely planted *H*. *brasiliensis* clone, RRIM 600. The genome was assembled based on ~155-fold combined coverage with Illumina and PacBio sequence data and has a total length of 1.55 Gb with 72.5% comprising repetitive DNA sequences. A total of 84,440 high-confidence protein-coding genes were predicted. Comparative genomic analysis revealed strong synteny between *H*. *brasiliensis* and other Euphorbiaceae genomes. Our data suggest that *H*. *brasiliensis*’s capacity to produce high levels of latex can be attributed to the expansion of rubber biosynthesis-related genes in its genome and the high expression of these genes in latex. Using cap analysis gene expression data, we illustrate the tissue-specific transcription profiles of rubber biosynthesis-related genes, revealing alternative means of transcriptional regulation. Our study adds to the understanding of *H*. *brasiliensis* biology and provides valuable genomic resources for future agronomic-related improvement of the rubber tree.

*Hevea brasiliensis* Muell. Arg, the Pará rubber tree, or most commonly known as the rubber tree, is a medium to large monoecious and cross-pollinated tropical tree belonging to the family Euphorbiaceae (spurge)^1^. This family of flowering plants encompasses more than 7,500 species spread across 300 genera of herbs, shrubs and trees. Besides *H. brasiliensis*, Euphorbiaceae includes a number of plants with economic importance, such as cassava (*Manihot esculenta*), the castor oil plant (*Ricinus communis*) and Barbados nut (*Jatropha curcas*). The genus *Hevea* is comprised of eleven species^2^, namely *H*. *brasiliensis*, *Hevea bethamiana*, *Hevea camporum*, *Hevea camargoana*, *Hevea guianensis*, *Hevea microphylla*, *Hevea nitida*, *Hevea pauciflora*, *Hevea paludosa*, *Hevea rigidifolia* and *Hevea spruceana*^3^,^4^,^5^. All *Hevea* species are diploid (2*n* = 2x = 36) with the exception of triploid *H*. *guianensis* (2*n* = 2x = 54) and the existence of one genotype of *H*. *pauciflora* with 2*n* = 2x = 18^6^,^7^. Although *Hevea* behaves as a diploid^8^, it is proposed that *Hevea* has an amphidiploid origin stabilized during the course of evolution^9^. Among the genus members, only *H*. *brasiliensis* yields an economically viable amount of latex that makes up most (99%) of the world’s natural rubber production. In 2014, global natural rubber production was estimated at 12 million tons with a corresponding value of 22 billion US dollars (http://www.lgm.gov.my).

The rubber tree is indigenous to the Amazon basin in South America, but is now widely planted in Southeast Asia, especially in Malaysia, Indonesia and Thailand. The domestication of rubber trees outside Brazil occurred more than 100 years ago with the transfer of *H*. *brasiliensis* seedlings to Asia^10^. *H*. *brasiliensis* is a deciduous tree that grows to 30–40 m tall and can live up to 100 years in the wild^11^. Natural rubber is a high molecular weight polymer made of repeating isoprene units in the *cis*-configuration. Natural rubber differs from synthetic rubber in polymer length and side chain composition, which contribute to its superior performance properties in elasticity, resilience and efficient heat dispersion. Hence, despite accounting for less than 50% of total rubber consumption, natural rubber is still indispensable for heavy-duty tires and high-performance engineering components.

In *H*. *brasiliensis*, rubber is synthesized on the surface of rubber particles suspended in the latex of laticifers or specialized parenchyma cells^12^. Six to seven years after planting, a rubber tree can be tapped for latex by controlled wounding of the bark tissue. *H*. *brasiliensis* clone RRIM 600, a secondary clone originally developed from clones Tijr 1 and PB 86, is the most widely planted clone in Malaysia and Thailand^13^,^14^. RRIM 600 is categorized as a medium yielding clone that produces ~1,350 kg of latex per hectare per year in estates over 20 years old. This clone shows moderate tolerance to cold, drought and wind, but is susceptible to disease caused by the pathogenic fungal *Phytophthora* spp. (http://www.rubberboard.org.in).

Despite its economic importance, past attempts in genetic analyses to improve *H*. *brasiliensis* rubber production and other desirable traits have been progressing slowly. A major hurdle for crop improvement of *H*. *brasiliensis* is the lack of a comprehensive whole-genome overview and therefore, a holistic knowledge of the latex biosynthetic process. A previous attempt to assemble the *H*. *brasiliensis* genome produced a fragmented draft using 43-fold sequence data^14^. A high-quality genome assembly for *H*. *brasiliensis* is imperative for discovery of the precise pathways and genes responsible for desirable agronomic traits. Here, we report a more complete *H*. *brasiliensis* genome assembly that allows genomic comparison with other sequenced plant genomes and cap analysis gene expression (CAGE) studies on tissue-specific transcription of genes, particularly those related to rubber biosynthesis. Our effort unraveled genome-level insights on the evolutionary relationship of *H*. *brasiliensis* with other Euphorbiaceae species. Most importantly, our analysis reveals possible contributors to *H*. *brasiliensis*’s capacity to produce high levels of latex and alternative means of transcriptional regulation for rubber biosynthesis.

## Results

### Genome sequencing and assembly

**Figure 1:**
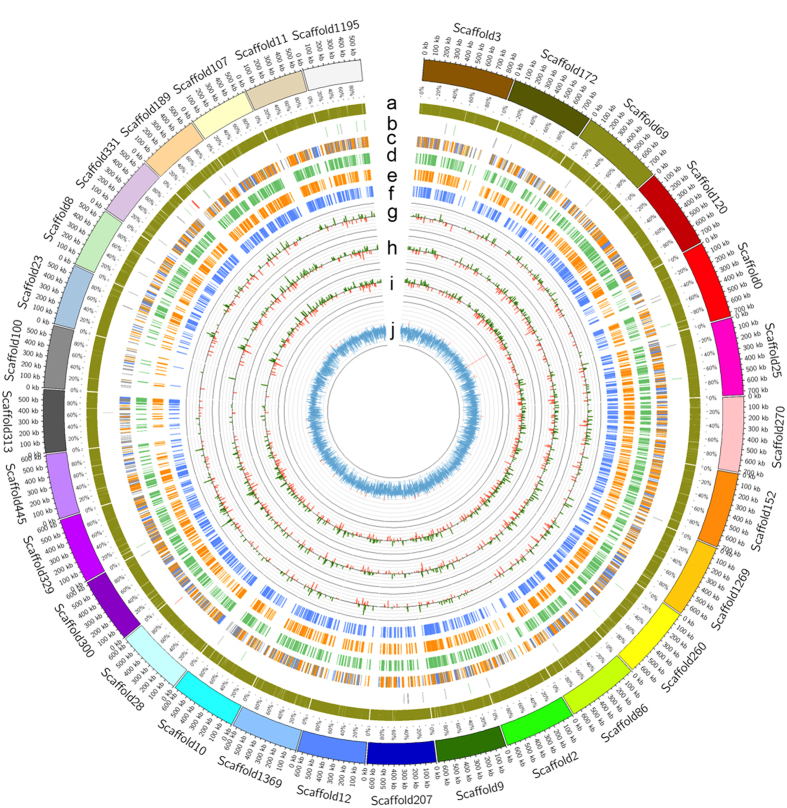
Characterization of the *H*. *brasiliensis* genome. Circos plot of the 30 longest scaffolds. (**a**) Repeats; (**b**) Non-coding RNAs (rRNAs, tRNAs and other ncRNAs are represented by red, green and grey lines, respectively); (**c**) Gene model annotation against the NCBI non-redundant protein database (BLAST matches to *R*. *communis*, *M*. *esculenta*, *J*. *curcas* and other organisms are represented by blue, green, orange and grey lines, respectively); (**d**) Orthologous genes in *M*. *esculenta*; (**e**) Orthologous genes in *J*. *curcas*; (**f**) Orthologous genes in *R*. *communis*; (**g**) CAGE tags per million (TPM) in latex (TSSs mapped to the sense and antisense strand are represented in green and red, respectively); (**h**) CAGE TPM in leaf (TSSs mapped to the sense and antisense strands are represented in green and red, respectively); (**i**) CAGE TPM in bark (TSSs mapped to the sense and antisense strands are represented in green and red, respectively) (**j**) GC content (values of >50% and < = 50% are represented in red and blue, respectively).

The genome assembly of *H*. *brasiliensis* was constructed using the whole-genome shotgun sequencing approach utilizing both Illumina and PacBio reads. A total of 288.84 Gb (~134-fold coverage) of raw sequencing data was generated using the Illumina platform from both paired-end and mate-pair libraries with different insert sizes (Supplementary Table S1). The genome was assembled using Platanus, a *de Brujin* graph-based assembler^15^. The size of the genome has been estimated to be 2.15 Gb based on 17-mer depth distributions of Illumina paired-end reads, and this is consistent with a previous estimate^16^ (Supplementary Figure S1). The Platanus assembly was further improved using long PacBio reads through the implementation of the PBJelly2 software^17^. A 10-Kb SMRTbell library was prepared and sequenced using 100 SMRT Cells, which yielded 45.25 Gb (~21-fold coverage) of sequence with a typical average read length of 6,852 bp (Supplementary Figure S2). The resulting final assembly spans 1.55 Gb with the largest scaffold being 871 Kb (Table 1). The genome assembly consists of 189,316 scaffolds with an N50 size of 67.2 Kb. Using high coverage sequence data and the application of PacBio long reads, the N50 size of our *H*. *brasiliensis* genome assembly was increased 23-fold and the number of scaffolds was decreased three-fold compared to the previously published assembly^14^. Our current *H*. *brasiliensis* genome assembly represents a great improvement from the previous one, and allows for detailed and accurate functional annotation. The assembly was anchored to 18 linkage groups based on the published linkage map^8^ (Supplementary Figure S3). In total, 189 scaffolds could be anchored to the genetic map through restriction fragment length polymorphism (RFLP) markers. The GC content of the assembled *H*. *brasiliensis* genome is 34.17%, similar to those of the sequenced genomes of *R*. *communis* (32.5%)^18^, *J*. *curcas* (33.7%)^19^ and *M*. *esculenta* (34.86%)^20^ from the Euphorbiaceae family (Fig. 1).

The completeness of the assembly was evaluated by aligning EST sequences of *H*. *brasiliensis* to the genome assembly. Of the 51,631 *H*. *brasiliensis* ESTs available from NCBI, 96.8% were detected in the assembly (Supplementary Table S2). The assembly was also validated by aligning assembled transcripts from RNA sequencing (RNA-seq) of different *H*. *brasiliensis* tissues^14^,^21^,^22^,^23^,^24^. More than 93.7% of the transcripts could be aligned to the assembly, indicating near-complete coverage of the gene space in the assembly. We also detected a total of 445 and 416 mitochondrial and chloroplast DNA fragment insertions into the nuclear genome covering 594,389 and 230,702 bp, respectively (Supplementary Table S3). In flowering plants, the transfer of DNA from the mitochondria or plastids to the nucleus has been identified^25^. In the *H*. *brasiliensis* genome, these insertions include three large mtDNA fragments (32–36 Kb).

The extent of genome duplications in *H*. *brasiliensis* was analyzed by aligning *H*. *brasiliensis* scaffolds against itself using reciprocal BLAST matches. The 90 pairs of scaffolds that contained the largest number of paralogous gene pairs were selected and visualized in a dotplot (Supplementary Figure S4). Although genome triplications that were identified in the *R*. *communis* genome^18^ were not observed, this approach identified a total of 47 duplicated regions.

### Repeat content of the *H*. *brasiliensis* genome

Transposable elements represent major constituents of plant genomes. A combination of *de novo* and homologue-based approaches resulted in the identification of 1,783,541 repetitive sequences, accounting for 72.5% of the assembled genome (Table 2). This level of repetitiveness is similar to those of the tobacco (72–79%)^26^, hot pepper (76.4%)^27^ and cultivated cotton (68.5%)^28^ genomes, but higher than those of castor bean (50.33%)^18^ and cassava (25.7–36.9%)^20^ from the Euphorbiaceae family (Supplementary Table S4). The noticeable proliferation of transposable elements in the *H*. *brasiliensis* genome has likely expanded its genome size compared to other Euphorbiaceae species. Overall, retrotransposons occupy the majority of the *H*. *brasiliensis* nuclear genome (~57.3%), of which ~11.7% and ~43.6% are Copia and Gypsy elements, respectively. In contrast, DNA transposons only represent ~1.2% of the nuclear DNA. Closer inspection revealed that the 3.71 ratio of Gypsy to Copia LTR-retrotransposons in the rubber genome is similar to that of the sorghum genome (3.66)^29^. The ratio of Gypsy to Copia elements in the genome generally varies between species^30^.

### Gene prediction and functional annotation

The Maker-P^31^,^32^ pipeline that combines *ab initio* gene predictions, homology-based searching and transcriptome alignments was used for genome annotation. Protein sequences from the *M*. *esculenta*, *R*. *communis*, *J*. *curcas*, *P*. *trichorpca* and Viridiplantae datasets from Swiss-Prot were used as homology-based evidence. Assembled contigs of RNA-seq reads from leaf, latex and bark tissues^21^ were used as transcript evidence. Only gene predictions that are supported by protein or transcript evidence are included in the final gene models. A total of 84,440 protein-coding genes were predicted, with an average transcript length of 3,069 bp, an average coding length of 971 bp and an average of 4.97 exons per gene. Among the predicted genes, 76.5% of the encoded proteins showed homology to sequences in the NCBI non-redundant protein database (Supplementary Table S5). Additionally, 39.7%, 74.7%, 56.6% and 72.8% of the genes could be functionally annotated using the Swiss-Prot, TrEMBL, GO and KEGG databases, respectively. GO annotation revealed that the *H*. *brasiliensis* genome maintains more genes associated with ‘binding’ and ‘catalytic activity’ subcategories than other genomes (Supplementary Figure S5). Furthermore, at least one conserved domain was detected in 65.5% of the predicted protein sequences by comparison against the InterPro database. In addition to protein-coding genes, non-coding RNAs in the assembly were also identified (Supplementary Table S6). A total of 739 transfer RNAs (tRNAs), 271 ribosomal RNAs (rRNAs), 192 spliceosomal RNAs, 231 small nucleolar RNAs and 206 micro RNAs were identified in the *H*. *brasiliensis* genome assembly.

### Comparative analyses between rubber tree and other plants

**Figure 2:**
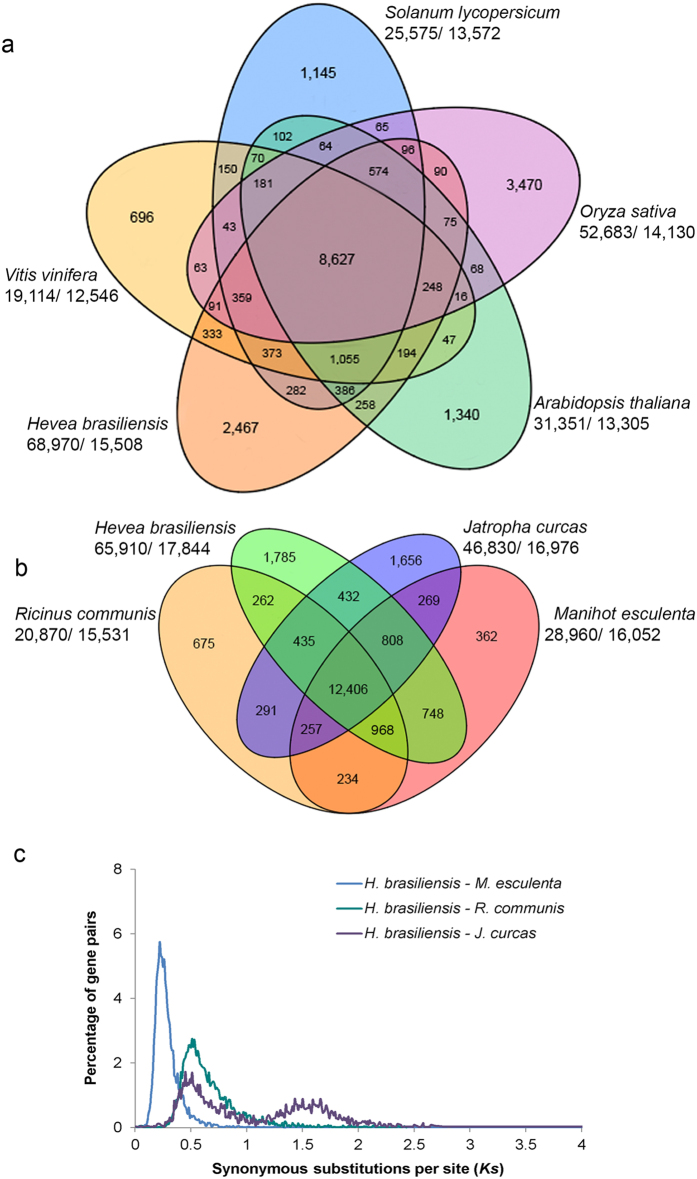
*H*. *brasiliensis* comparative genomics. Venn diagrams show unique and shared gene families between and among representative (**a**) angiosperms and (**b**) Euphorbiaceae. The number of genes in the families and the number of gene families are indicated under each species name. (**c**) Distribution of synonymous nucleotide substitution (*Ks*) rates. The blue, turquoise and purple lines represent the *Ks* distribution of orthologous gene pairs in *H*. *brasiliensis*-*M*. *esculenta*, *H*. *brasiliensis*-*R*. *communis* and *H*. *brasiliensis*-*J*. *curcas*, respectively. 8,136, 7,399 and 3,456 single-copy orthologs, were used for the *H*. *brasiliensis*-*M*. *esculenta*, *H*. *brasiliensis*-*R*. *communis* and *H*. *brasiliensis*-*J*. *curcas* analyses, respectively.

**Figure 3:**
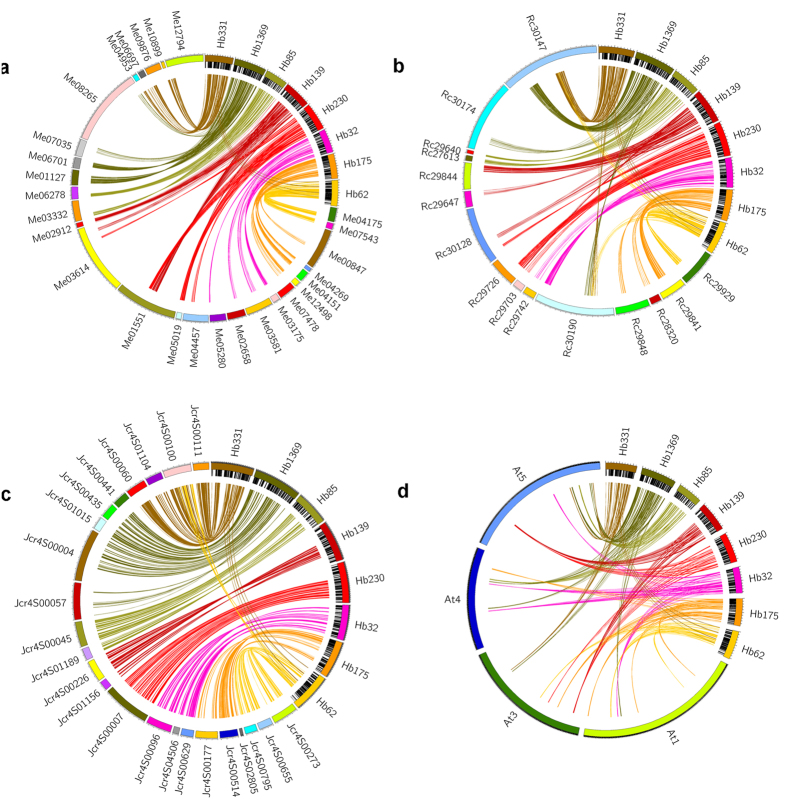
Macro-synteny between *H*. *brasiliensis* (Hb) and (**a**) *M*. *esculenta* (Me), (**b**) *R*. *communis* (Rc), (**c**) *J*. *curcas* (Jcr) and (**d**) *A*. *thaliana* (At). Labels around the circles correspond to scaffold numbers in *H*. *brasiliensis*, *M*. *esculenta*, *R*. *communis*, and *J*. *curcas*, and chromosome numbers in *A*. *thaliana*.

**Figure 4:**
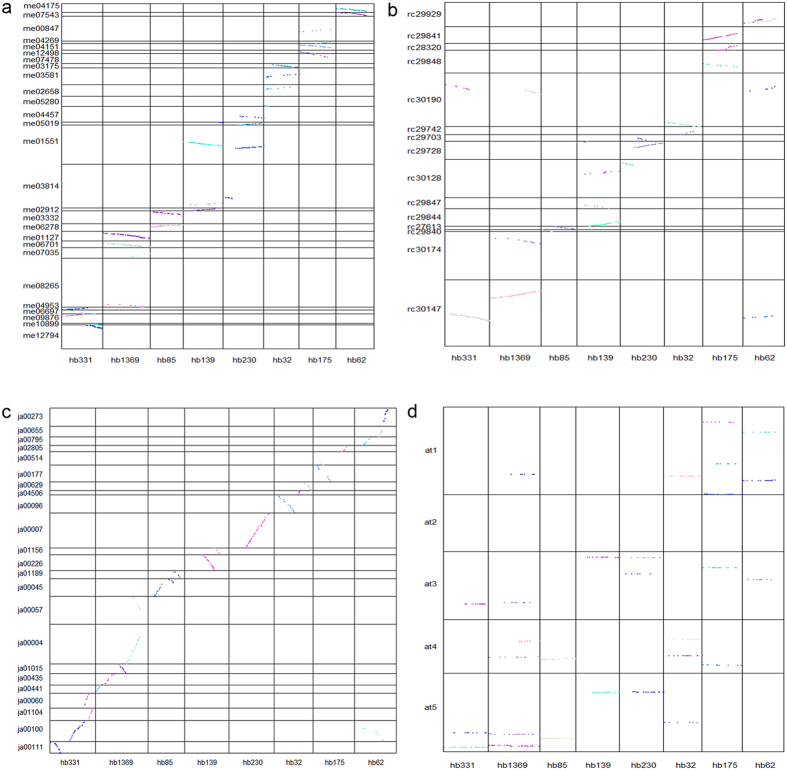
Dot-plot analyses between *H*. *brasiliensis* and (**a**) *M*. *esculenta*, (**b**) *R*. *communis*, (**c**) *J*. *curcas* scaffolds and (**d**) *A*. *thaliana* chromosomes. Each dot represents a collinear gene pair between *H*. *brasiliensis*, *M*. *esculenta*, *R*. *communis*, *J*. *curcas* and *A*. *thaliana* (BLASTP *e*-value cutoff < 10^−5^). Different coloured dots represent different collinear blocks. Eight scaffolds of *H*. *brasiliensis* that have more than 15 homologous genes in *M. esculenta, R. communis, J. curcas*, and *A. thaliana* are shown.

The *H*. *brasiliensis* genome contains similar patterns of orthologous gene sets in comparison to representative species from monocotyledons (*Oryza sativa*) and dicotyledons (*Arabidopsis thaliana*, *Vitis vinifera* and *Solanum lycopersicum*), sharing a total of 8,627 common gene families (Fig. 2a). Proteome comparison between *H*. *brasiliensis* and other species from Euphorbiaceae (*R*. *communis*, *M*. *esculenta* and *J*. *curcas*) revealed that a large cluster of 12,406 gene families is shared among the four Euphorbiaceae genomes (Fig. 2b). The 1,785 gene families that are unique to *H*. *brasiliensis* are significantly enriched with genes related to molecular function categories such as ‘zinc ion binding’, ‘cation binding’ and ‘transition metal ion binding’ (Supplementary Table S7). Of the Euphorbiaceae species with sequenced genomes, *M*. *esculenta* is the closest relative of *H*. *brasiliensis*. Consecutively, the largest number of gene clusters from 14,930 gene families is shared between *H*. *brasiliensis* and *M*. *esculenta* compared to the other plants examined in this study. *Ks* plots show that *H*. *brasiliensis* and *M*. *esculenta* diverged later than the divergence of *R*. *communis* and *J*. *curcas* in the Euphorbiaceae family (Fig. 2c). The macro-synteny between the genome of *H*. *brasiliensis* and those of distantly related species (*R*. *communis*, *M*. *esculenta*, *J*. *curcas*), as well as the more distantly diverged species (*A*. *thaliana*), was evaluated (Fig. 3). We found strong synteny between *H*. *brasiliensis* and other Euphorbiaceae species, but much weaker synteny between *H*. *brasiliensis* and *A*. *thaliana*. Alignment of *H*. *brasiliensis* scaffolds to their best-matched *M*. *esculenta*, *R*. *communis*, *J*. *curcas* and *A*. *thaliana* genomic regions was performed to analyze intergenome collinearity between these genomes (Fig. 4). On average, *H*. *brasiliensis* gene blocks showed one-to-one, one-to-two and one-to-three synteny relationships with their orthologues in *J*. *curcas*, *M*. *esculenta* and *R*. *communis*, respectively. Comparison between *H*. *brasiliensis* and *A*. *thaliana* revealed that many regions of the *H*. *brasiliensis* genome have two, but some have three or more, homologous regions in *A*. *thaliana*.

### Transcription factor families

Within the *H*. *brasiliensis* genome, 3,142 putative transcription factors were classified into 89 subfamilies (Supplementary Table S8). Despite having a larger genome size, the core set of transcription factors in *H*. *brasiliensis* does not significantly differ from other plants. *H. brasiliensis* showed a very similar distribution of transcription factor families to *A. thaliana, S. lycopersicum*, *O. sativa* and *R. communis*. In comparison with these other plants, *H. brasiliensis* and *O. sativa* both have three times more FAR1 transcription factors in their genomes. FAR1 is related to a mutator-like transposase and is involved in modulation of phyA-mediated light signaling in *A. thaliana*^33^. The high frequency of this family in the genome may indicate a more diverged role of this transcription factor in *H*. *brasiliensis* and *O*. *sativa*. Overall, MYB constitutes the most abundant group of transcription factors in *H*. *brasiliensis*. These particular groups of transcription factors are important regulators of plant development, including hormone signal transduction, disease resistance and secondary metabolism^34^.

### Disease resistance

Disease resistance is one of the most important traits identified as a target for rubber plant breeding. The rubber tree is susceptible to several fungal diseases, including South American leaf blight (*Microcyclus ulei*), abnormal leaf fall (*Phytopthora* spp.), powdery mildew (*Oidium heveae*), *Corneyspora* leaf fall (*Cornyespora cassiicola*), pink disease (*Corticium salmonicolor*) and white root disease (*Rigidoporus* spp.)^2^. Genes related to disease resistance (R genes) play an important role in the resistance mechanism of plants, and information on these genes in the *H*. *brasiliensis* genome will provide a basis for improvement of disease resistance.

We identified 483 disease resistance-related genes, which is the highest among the four Euphorbiaceae plants examined here (Supplementary Table S9). This expansion of R genes in *H*. *brasiliensis* suggests an enhanced ability to detect and defend against pathogen invasion in latex-producing *H*. *brasiliensis*. These R genes found in the *H*. *brasiliensis* genome belong to the nucleotide-binding site (NBS) resistance genes and constitute about 0.57% of all *H*. *brasiliensis* genes. The NBS-encoding genes can be classified into multiple subfamilies, including 186 NBS-leucine-rich repeat (NBS-LRR), 45 coiled-coil (CC)-NBS-LRR, 35 CC-NBS, 15 Toll interleukin-1 receptor-NBS-LRR (TIR-NBS-LRR) and 4 TIR-NBS. Some of these disease resistance-related genes were found organized in clusters in the *H*. *brasiliensis* genome. In particular, scaffold284, scaffold589 and scaffold2863 were found to contain clusters containing four, six and four disease resistance-related genes, respectively (Supplementary Figure S6). These observations are consistent with previous classical genetic and molecular data which reports that plant disease resistance genes are often clustered in the genome^35^.

The plant defense response is regulated through a complex network of signal transduction pathways involving three signaling molecules: salicylic acid (SA), ethylene and jasmonic acid (JA). Nonexpressor of PR genes 1 (NPR1) and its paralogues, NPR3 and NPR4, are known to function as SA receptors^36^,^37^. JA is also involved in laticifer cell differentiation, and coronatine insensitive 1 (COI1) has been identified as its receptor^38^. In the *H*. *brasiliensis* genome, the *COI1*, *NPR1* and *NPR3* genes occur in duplicate pairs and are distributed across different scaffolds (Supplementary Table S10). In contrast, NPR4 is encoded by a single-copy gene. Besides functioning as a defense signaling component, the gaseous phytohormone ethylene is an essential element in regulating rubber tree latex production^39^. The ethylene receptor, ETR1, is encoded by four genes in *H*. *brasiliensis*. Most of the disease resistance signaling components contain multiple homologues in *H*. *brasiliensis*, indicating the importance of these genes in plant defense.

### Isoprenoid biosynthesis

Natural rubber from *H*. *brasiliensis* is a high molecular weight biopolymer that is made up of *cis*-isoprene units derived from isopentenyl diphosophate (IPP). The biosynthesis of IPP proceeds via two distinct routes: the cytoplasmic mevalonate pathway (MVA) and the plastidic 2-C-methyl-_D_-erythritol 4-phosphate (MEP) pathway (Supplementary Figure S7). In the former, IPP is derived from the condensation of three acetyl-CoA moieties; in the MEP pathway, IPP is formed from the condensation of pyruvate and _D_-glyceraldehyde 3-phosphate. Radiolabelling of the intermediates of the MVA pathway provided evidence that IPP synthesized from the MVA pathway is utilized for *cis*-polyisoprene production^40^,^41^. The MEP pathway has also been considered as a possible alternative means of generating IPP for rubber biosynthesis because the expression of 1-deoxy-_D_-xylulose-5-phosphate synthase (DXP synthase) was observed in *H*. *brasiliensis* latex and leaves^42^. From our annotation, the *H*. *brasiliensis* genome has a larger number of MVA and MEP pathway genes than *A*. *thaliana* (58 versus 33). For example, DXP synthase, catalyzing the first step in the MEP pathway, has expanded from three genes in *A*. *thaliana* to nine in *H*. *brasiliensis*. All of the enzymes involved in the MVA and MEP pathways are encoded by multiple genes in *H*. *brasiliensis*, indicating the expansion of these gene families in this genome. The expansion of MVA and MEP pathway genes in *H*. *brasiliensis* meets the need for its formation of isoprenoids.

### Rubber biosynthesis

**Figure 5:**
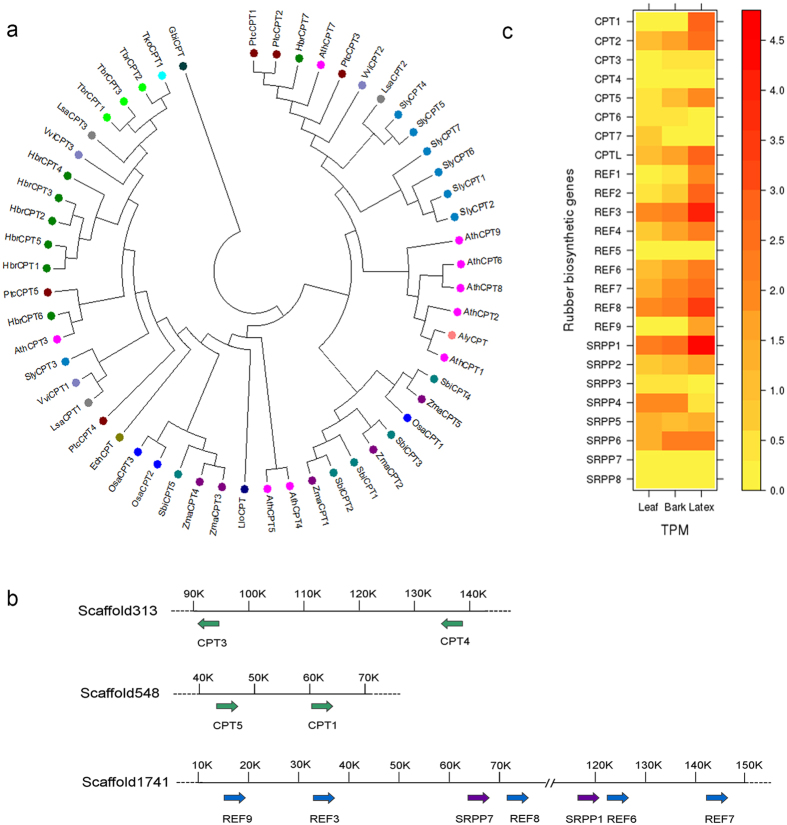
*H*. *brasiliensis* rubber biosynthesis-related genes. (**a**) Phylogenetic analysis of the CPT protein family based on the maximum-likelihood method using MEGA (version 6.06). Three letter codes: Ath, *A*. *thaliana*; Aly, *Arabidopsis lyrata*; Ech, *Euphorbia characias*; Gbi, *Ginkgo biloba*; Hbr, *H*. *brasiliensis*; Lsa, *Lactuca sativa*; Llo, *Lilium longiflorum*; Osa, *O*. *sativa*; Ptc, *Populus trichocarpa*; Sbi, *Sorghum bicolor*; Sly, *S*. *lycopersicum*; Tbr, *Taraxacum brevicorniculatum*; Tko, *Taraxacum kok-saghyz*; Vvi, *V*. *vinifera*; Zma, *Zea mays*. (**b**) Organization of rubber biosynthesis-related gene clusters across the genome. (**c**) Heat map of the normalized CAGE expression level for genes associated with rubber biosynthesis. The heat map was plotted using the Lattice (version 0.29.33) package in R.

Latex production is an important biological feature of the rubber tree that has tremendous economic importance. Natural rubber is synthesized by the successive addition of IPP molecules to a priming allylic diphosphate (farnesyl diphosphate and/ or geranylgeranyl diphosphate)^43^. The enzymes leading to the formation of allylic diphosphate from IPP are encoded by multiple genes (isopentenyl-diphosphate delta isomerase, dimethyallyl transferase, farnesyl diphosphate synthase and geranyl geranyl diphosphate synthase) in *H*. *brasiliensis* (Supplementary Table S11). The polymerization of *cis*-1,4-polyisoprene is mediated by a membrane-bound rubber transferase complex with *cis*-prenyltransferase (CPT) activity^44^. In *H*. *brasiliensis*, the entire CPT gene family comprising seven members (designated as CPT1-7) was identified. Based on the phylogenetic analysis of CPT sequences from *H*. *brasiliensis* and 14 other plant species, five of the seven *H*. *brasiliensis* CPT genes (CPT1-5) form a subgroup (Fig. 5a). A member from this cluster (CPT2) was shown to exhibit *in vitro* long-chain prenyltransferase activity in the presence of washed latex particles^45^.

The two most abundant rubber particle proteins, rubber elongation factor (REF) and small rubber particle (SRPP), have been shown to participate in *cis*-polyisoprene production^45^. In *H*. *brasiliensis*, REF and SRPP are encoded by nine and eight genes, respectively, which are the largest numbers reported in any plant genome (Supplementary Figure S8). This significant expansion of rubber biosynthesis-related genes in the *H*. *brasiliensis* genome correlates with its capacity to produce high levels of latex. A *cis*-prenyltransferase-like (CPTL) protein that was suggested to be involved in rubber biosynthesis in lettuce and dandelion^46^,^47^ was also found in the *H*. *brasiliensis* genome. Among the annotated rubber biosynthesis-related genes, some are found in gene clusters (Fig. 5b). For example, clusters containing two CPT genes that were likely generated by tandem duplication are distributed along scaffold313 and scaffold548. A larger gene cluster containing seven rubber particle proteins is located on scaffold1741. In addition, SRPP7 and REF8, and SRPP1 and REF6 are arranged in a tandem array on scaffold1741. Since both gene pairs are phylogenetically close, they may have derived from a tandem duplication event.

### CAGE analysis

To understand the transcriptional control of *H*. *brasiliensis* genes, we analyzed the transcription start sites (TSSs) of genes expressed in different tissues by CAGE analysis. This method enables high-throughput gene expression analysis and the precise mapping of TSSs to the genome^48^. Unique tags that start within the tag cluster are grouped into CAGE-tag starting sites (CTSSs), whereas overlapping CTSSs on the same strand form a tag cluster (TC)^49^. In total, 484 million CAGE tags were sequenced based on libraries prepared from total RNAs isolated from bark, latex and leaf (Supplementary Table S12). Mapping of the CAGE tags to the *H*. *brasiliensis* genome allowed us to identify 2,948,134 potential TSSs. The CAGE data obtained from biological replicates derived from different tissues showed high correlation, indicating the reproducibility of the analysis (Supplementary Figure S9). The location of the mapped CAGE tags was examined (Supplementary Table S13). In total 31,033 TCs were identified. Approximately 34% (10,786) of the CAGE tags mapped at most 2,000 bp upstream of the transcripts, allowing for the identification of new TSSs. Accordingly, 54% (17,030) and 11% (3,552) of the CAGE tags mapped inside genes or within the intergenic regions, respectively.

Comparative analysis between bark, latex and leaf gene expression data revealed tissue-specific expression related to the function of these tissues. From the 31,033 total TCs identified in *H*. *brasiliensis*, 1,136 were found only in latex, 4,158 in bark and 5,516 in leaf (Supplementary Figure S10). GO analysis of the transcripts expressed only in latex revealed an enrichment in genes involved in cellular components including ‘cell’, ‘intracellular’, ‘organelle’ and ‘membrane-bounded organelle’ categories, which is consistent with the fact that latex is the cytoplasmic content of the laticifer cells (Supplementary Table S14). In bark-specific transcripts, genes belonging to the GO categories ‘transcription factor activity, sequence-specific DNA binding’, ‘catalytic activity’ and ‘cell wall organization’ are enriched (Supplementary Table S15). The enrichment of GO categories ‘chloroplast thylakoid membrane’, ‘pentose-phosphate shunt’ and ‘photosystem II’ that are related to photosynthesis was observed in leaf-specific transcripts (Supplementary Table S16).

### CAGE profiling reveals tissue-specific transcription

**Figure 6:**
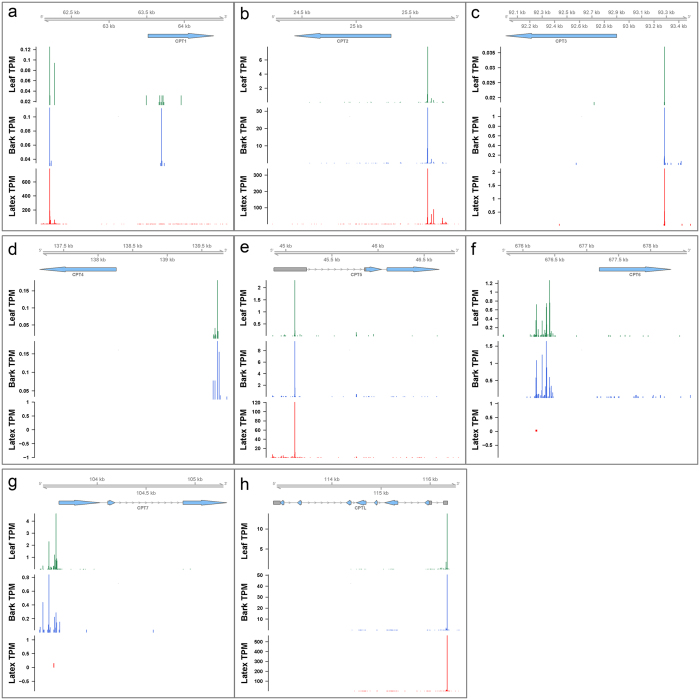
CAGE tag cluster distribution for (**a–g**) CPT1-7 and (**h**) CPTL in leaf, bark and latex. The top arrows represent Maker-P predicted gene structures; grey boxes are 5′/3′ UTR and blue arrows are CDSs. The histograms show normalized expression (TPM) and position of TSSs. Each histogram has a different scale for TPM. The relative locations of each gene in the scaffold are indicated at the top of the figure.

The expression profiles of rubber biosynthesis-related genes including CPTs, CPTL, SRPPs and REFs in different tissues were analyzed to investigate the genetic basis underlying latex production (Fig. 5c). It was found that CPT1-CP3, CPT5, CPTL, REF1-4, REF6-9, SRPP1-2 and SRPP4-6 were expressed in all the tissues (bark, latex and leaf) examined here (Fig. 6, Supplementary Figures S11–12). Although these key genes associated with rubber biosynthesis were expressed in multiple tissues, the highest expression levels were observed mostly in latex, suggesting the involvement of these genes in long chain *cis*-polyisoprene production. Their high expression levels most likely correlate with the high latex yield in *H*. *brasiliensis*. In contrast, CPT4 and CPT6-7 are expressed only in bark and leaf, indicating the possible role of these CPTs in short-chain prenyl product synthesis. Most CPTs, REFs and SRPPs show single dominant peak TSSs except for CPT1, REF1, REF4, REF9, SRPP2, SRPP5 and SRPP8 that have multimodal shapes of TSSs. CAGE-based TSS profiles indicate that some of these rubber biosynthesis-related genes (CPT1, CPT7, REF4, SRPP2 and SRPP8) have different TSSs depending on the tissue. The observation of multiple TSSs from the CAGE analysis provides invaluable resources for identifying alternative promoters that cannot be obtained by other transcriptomic methods.

The expression profiles of MVA and MEP pathway genes were also examined to provide insights into the formation of the intermediates associated with rubber biosynthesis. CAGE analysis revealed high expression levels of MVA pathway genes in latex, as compared to leaf and bark (Supplementary Figure S13). One member of the acetyl-CoA acetyltransferase, HMG-CoA synthase and mevalonate diphosphate decarboxylase gene families was highly expressed in latex. In contrast, MEP pathway genes were not preferentially expressed in latex. These observations revealed that the MVA pathway is the main contributor to the formation of the intermediates required for latex production and the enzymes discussed above may be the rate-limiting enzymes for high latex yield.

## Discussion

The *H*. *brasiliensis* genome was characterized as high in repeat content and heterozygosity, and with a large genome size that poses technical challenges in assembling its genome. Using a combination of a high coverage Illumina paired-end, mate-pair and long PacBio reads, the *H. brasiliensis* RRIM 600 genome was assembled. The final assembly spans 1.55 Gb and represents more than 93.7% of the transcriptome sequences^14^,^21^,^22^,^23^,^24^ and ESTs from publicly available databases. The current *H*. *brasiliensis* genome assembly is much improved in continuity, allowing for comparative analyses with other plant genomes and genome-wide characterization of protein-coding genes.

Comparative genomics analysis clearly indicates conserved synteny between *H*. *brasiliensis* and other members of Euphorbiaceae including *M*. *esculenta*, *R*. *communis* and *J*. *curcas*^18^,^19^,^20^. In most cases, *H*. *brasiliensis* gene blocks showed one-to-one, one-to-two and one-to-three synteny relationships with *J*. *curcas*, *M*. *esculenta* and *R*. *communis*, respectively. When compared with other Euphorbiaceae genomes, our analysis revealed that *H*. *brasiliensis* has the highest number of disease resistance-related genes. The expanded inventory of these genes could conceivably play a role in resistance to abiotic stresses or diseases caused by fungal pathogens. Our genomic and CAGE-seq analyses offer some insights into the biological features that are unique to *H*. *brasiliensis*, particularly those that are involved in rubber biosynthesis. The *H*. *brasiliensis* genome contains one of the richest set of rubber biosynthesis-related genes including CPTs, REFs and SRPPs. The expansion of these gene families and the high expression of these genes in latex could be an important contributor to high latex production in *H*. *brasiliensis*. Interestingly, some of these rubber biosynthesis-related genes are organized in clusters in the genome, indicating coordinated evolution and expression for latex production. Expression profiles of rubber biosynthesis-related genes analyzed using CAGE analysis showed that some of these genes exhibit tissue-specific TSSs. Although further studies are required to confirm these observations, these results reveal alternative means of transcriptional regulation besides changes in mRNA abundance. Detailed analyses on the expression of these rubber biosynthesis-related genes and their complex transcriptional control provide clues to understanding the unique properties of the rubber tree. The whole-genome sequence information and CAGE-seq data are valuable resources for use in the improvement of the economically important traits of the rubber tree.

## Methods

### DNA preparation and genome sequencing

High-quality genomic DNA was extracted from young leaves of *H*. *brasiliensis* clone RRIM 600 using the Qiagen DNeasy Plant Mini kit (Qiagen, Germany) following the manufacturer’s instructions. A 500 bp insert size paired-end library was prepared using the Illumina TruSeq DNA Sample Preparation Kit version 2 (Illumina, USA). Mate-pair libraries with insert sizes of ~3 Kb, 5 Kb, 7 Kb and 10 Kb were prepared according to the Illumina Nextera Mate Pair Sample Prep Kit (Illumina, USA). Sequencing was performed on the Hiseq 2500 and Miseq systems (Illumina, USA). One PacBio library with a 10 Kb insert size was sequenced with 100 SMRT cells using P5-C3 chemistry. PacBio sequencing was performed on RS II (Pacific Biosciences, USA) at the Yale Center for Genomic Analysis in West Haven, CT, USA.

### RNA preparation and CAGE analysis

Total RNAs were isolated from leaf, bark and latex of *H*. *brasiliensis* clone RRIM 600. Leaf and bark samples were obtained from young rubber plants; latex was collected from mature plants growing at the Centre for Chemical Biology, Penang, Malaysia. Fresh tissues from leaves and bark were flash-frozen in liquid nitrogen and ground to a fine powder. Latex samples were added to the extraction buffer directly. High-quality total RNAs were extracted using the CTAB-LiCl reagent followed by further TRI Reagent® (MRC, USA) treatment^50^. The quality of the purified RNA was confirmed using a Bioanalyzer 2100 (Agilent Technologies, USA). CAGE libraries were prepared according to the protocol described previously^51^ and sequenced on a HiSeq 2500 system. For quality control of the CAGE sequencing data, the TagDust2^52^ program was used to remove data artifacts. For mapping of the CAGE reads to the genome, BWA (version 0.7.12-r1039)^53^ was used. TSS clustering was performed using RECUL (version 1.0)^54^, and finally CAGEr (version 1.10.3)^55^ was used to analyze the TSSs.

### Genome assembly

Raw sequencing reads from each library (Supplementary Table S1) were preprocessed with the program Quake (version 0.3)^56^. Adaptor sequences in the mate-pair reads were trimmed using NextClip (version 1.3)^57^. The paired-end reads were then assembled into contigs using Platanus version 1.2.1 with *k*-mer auto-extension^15^. Reads from paired-end and mate-pair libraries were used for scaffolding by increasing library size. Gaps within the scaffolds were filled using the gap-close step in Platanus. Finally, PacBio reads were used to improve the genome assembly with PBJelly version 14.9.9^17^. The PacBio reads were mapped to the assembly using BLASR (version 1.3.1.127046) with parameters: minMatch = 8, sdpTupleSize = 8, minPctIdentity = 75, bestn = 1, nCandidates = 10, maxScore = 500 and noSplitSubreads. To anchor the assembled scaffolds onto 18 pseudo-chromosomes, 196 RFLP markers from the published genetic map^8^ were mapped to the assembly using BLASTN at an *e*-value cutoff of 1*e*-20. Hits that comprised > 90% identity and > 85% coverage were considered mapped markers. Quality of the genome assembly was assessed by mapping all available *H*. *brasiliensis* EST sequences downloaded from NCBI and published transcriptome data from leaf, latex and bark tissues^14^,^21^,^22^,^23^,^24^ using BLASTN at a threshold of 90% identity.

### Repeat content

Repeat sequences in the genome assembly were identified using RepeatMasker (version 4.0.5)^58^ with RepBase^59^ and a custom repeat library generated using RepeatModeler (version 1.0.8). RepeatModeler uses Tandem Repeats Finder (version 4.04)^60^ to search for tandem repeats in the genome and two programs, RECON (version 1.08)^61^ and RepeatScout (version 1.0.5)^62^, for *de novo* identification of repeat families in the genome sequences.

### Gene prediction and annotation

Gene models were predicted using the Maker-P pipeline (version 2.31.8)^31^,^32^. The gene prediction pipeline combined *ab initio* gene predictions, homology-based searching and transcriptome alignment. Augustus (version 3.0.3)^63^, Snap (version 2013-11-29)^64^, Fgenesh (version 2.6)^65^ and GeneMark-ES (version 4.21)^66^ were used for *de novo* gene prediction. Protein sequences from *M*. *esculenta*, *R*. *communis*, *J*. *curcas*, *P*. *trichorpca* and the Viridiplantae dataset from Swiss-Prot were used as homology-based evidence. RNA-seq reads from leaf, latex and bark tissues^21^ were assembled using Oases (version 0.2.8)^67^ and used as transcript evidence. Maker-P was first run using the est2genome = 1 option to infer gene models directly from transcript evidence. The obtained gene models were then used to train the gene predictors, Augustus and Snap. Maker-P was run iteratively two additional times and the results from one run were used to train gene predictors that were used during the next run. Functional annotation of the predicted gene models was based on comparison with the NCBI, TrEMBL, Swiss-Prot and KEGG databases with a minimal *e*-value of 1*e*-5. GO terms were assigned to the annotated genes using the Blast2GO pipeline (version 3.1.3)^68^. Protein domains and functions were analyzed using InterProScan (version 5.13–52.0)^69^. The tRNAs were identified using tRNAscan-SE (version 1.3.1)^70^; rRNAs, snRNAs, snoRNAs and microRNAs were identified by searching the genome assembly against the Rfam database (Release 12.0)^71^ using Infernal (version 1.1.1)^72^ with an *e*-value cutoff of 1*e*-10. Transcription factors in *H*. *brasiliensis* were identified and classified into families using the iTAK pipeline (http://bioinfo.bti.cornell.edu/tool/itak).

### Comparative analysis

The non-redundant protein dataset from *H*. *brasiliensis*, *A*. *thaliana*, *O*. *sativa*, *S*. *lycopersicum*, *V*. *vinifera*, *M*. *esculenta*, *R*. *communis* and *J*. *curcas* was used to identify gene clusters. All-against-all BLASTP analysis of all protein sequences was performed with an *e*-value cutoff of 1*e*-5. OrthoMCL (version 2.0.9)^73^ was used to construct gene families with a default inflation value of 1.5. Synonymous (*Ks*) and non-synonymous (*Ka*) substitution rates for orthologous gene pairs in *H*. *brasiliensis* and Euphorbiaceae members were computed using the KaKs_Calculator tool (version 2.0)^74^. Syntenic blocks and gene collinearity were inferred using MCScanX (version 2013-11-11)^75^. *H*. *brasiliensis*’s scaffolds that have more than 15 gene syntenic regions against all four organisms examined (*M*. *esculenta*, *R*. *communis*, *J*. *curcas* and *A*. *thaliana*) were visualized using Circos (version 0.67–7).

### Identification of disease resistance genes

NBS genes in *H*. *brasiliensis* were identified using HMMER (version 3.1)^76^ search analysis to screen the predicted proteome against the raw hidden Markov model (HMM) corresponding to the Pfam NBS (NB-ARC) family domain. The TIR and LRR domains in the predicted NBS-encoding amino acid sequences were screened using HMMER search analysis against the HMM model Pfam TIR and LRR domains, respectively. CC motifs were analyzed using Paircoil2^77^ with a P-score cutoff of 0.025.

## Additional Information

**Accession codes:** The *H. brasiliensis* genome assembly, genome and CAGE sequencing data were deposited at DDBJ/EMBL/GenBank BioProject under accession PRJDB4387. The rubber tree genome database is accessible at http://plant.psc.riken.jp/cgi-bin/gb2/gbrowse/rubber.

**How to cite this article**: Lau, N.-S. *et al*. The rubber tree genome shows expansion of gene family associated with rubber biosynthesis. *Sci. Rep*. **6**, 28594; doi: 10.1038/srep28594 (2016).

## Supplementary Material

Supplementary Information

## Figures and Tables

**Table 1:** Statistics of the *H*. *brasiliensis* genome assembly.

Feature	Value
Number of scaffolds	189,316
Longest scaffold (Kb)	871.19
N50 scaffold length (Kb)	67.24
Total size of scaffolds (Gb)	1.55
Number of contigs	262,709
N50 contig length (Kb)	20.75
Longest contig (Kb)	325.50
Total size of all contigs (Gb)	1.51
GC content (%)	34.17
Number of predicted protein-coding gene models	84,440
Average transcript length (bp)	3,069
Average number of exons per gene	4.97
Average coding sequence length (bp)	971
Average exon length (bp)	196
Average intron length (bp)	478

**Table 2:** Repeat element analysis in the *H*. *brasiliensis* genome.

Repeat elements	Copies (number)	Length occupied (bp)	Genome (%)
Retrotransposon	881,720	888,699,356	57.310
SINE	367	43,121	0.003
LINE	25,098	17,662,094	1.139
LTR/Copia	184,681	181,996,743	11.737
LTR/Gypsy	649,589	675,583,092	43.567
LTR/others	21,985	13,414,306	0.865
DNA transposon	48,232	18,463,525	1.191
Unknown	461,056	194,823,043	12.564
Satellites	234	34,261	0.002
Simple repeats	326,501	18,738,351	1.211
Low complexity	65,798	3,632,769	0.235
**Total**	1,783,541	1,124,391,305	72.510
