# Supplementary material for: The rubber tree genome shows expansion of gene family associated with rubber biosynthesis
Source: Sci Rep. 2016 Jun 24;6:28594. doi: 10.1038/srep28594 (PMC5008842; doi:10.1038/srep28594)
Supplement: Supplementary Information [file srep28594-s1.pdf]

The rubber tree genome shows expansion of gene family associated with rubber biosynthesis

Nyok-Sean Lau[1,2], Yuko Makita[2], Mika Kawashima[2], Todd D. Taylor[3], Shinji Kondo[4], Ahmad Sofiman Othman[1,5], Alexander Chong Shu-Chien[1,5,*], Minami Matsui[2,*]

[1] Centre for Chemical Biology, Universiti Sains Malaysia, Bayan Lepas, Penang, Malaysia.

[2] Synthetic Genomics Research Group, RIKEN Center for Sustainable Resource Science, Biomass Engineering Research Division, Tsurumi, Yokohama, Kanagawa, Japan.

[3] Laboratory for Integrated Bioinformatics, RIKEN Center for Integrative Medical Sciences, Tsurumi, Yokohama, Japan.

[4] Transdisciplinary Research Integration Center, National Institute of Polar Research, Tokyo, Japan.

[5] School of Biological Sciences, Universiti Sains Malaysia, Minden, Penang, Malaysia.

*Correspondence: alex@usm.my; minami@riken.jp

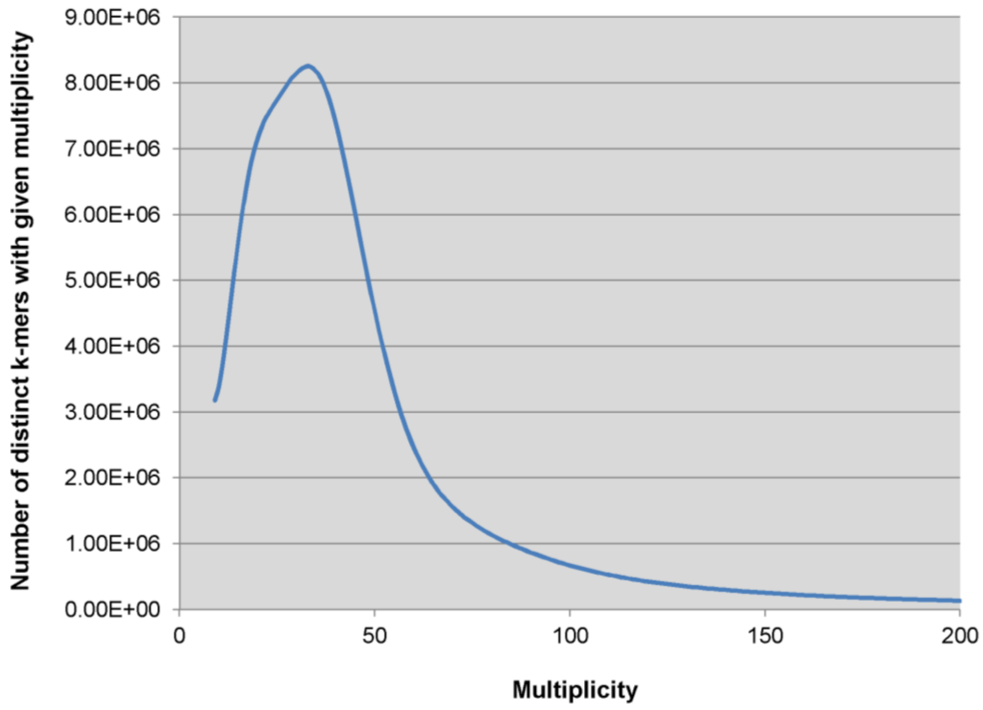

Supplementary Figure 1. Distribution of 17-mer frequency in the *H. brasiliensis* genome sequencing reads. The frequency of each 17-mer was calculated based on the preprocessed paired-end Illumina reads with insert size of 500 bp. The genome size was estimated as $(N\times(L-K+1)-B)/D=G$, where N is the total number of sequence reads, L is the length of the sequence reads, K is the K-mer length, B is the total number of low-frequency (frequency $\leq$ 1) K-mers, G is the genome size and D is the K-mer depth.

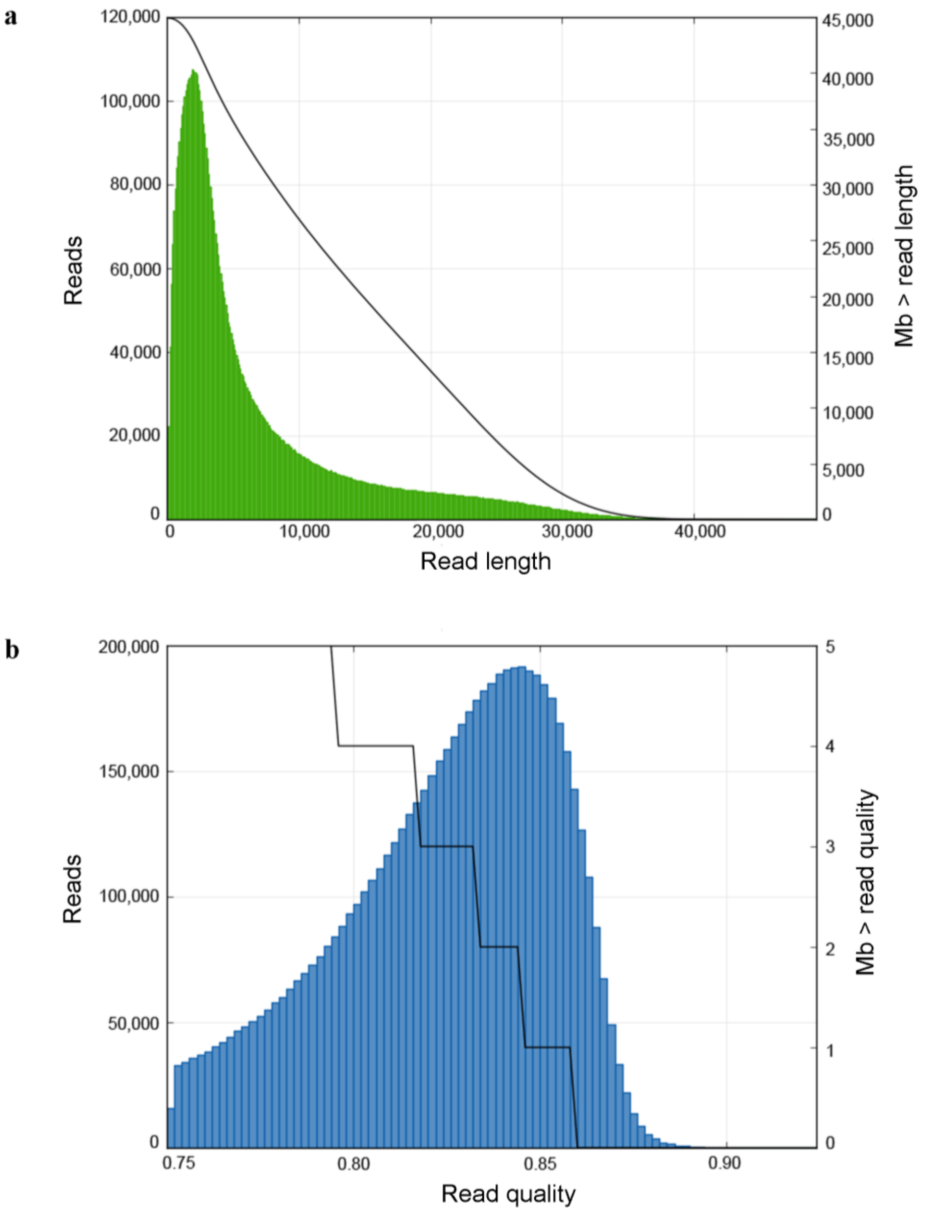

Supplementary Figure 2. (a) Length and (b) quality distribution of PacBio reads. 100 SMRT Cell runs generated 6,603,402 reads with an average length of 6.8 Kb totalling 45.25 Gb data.

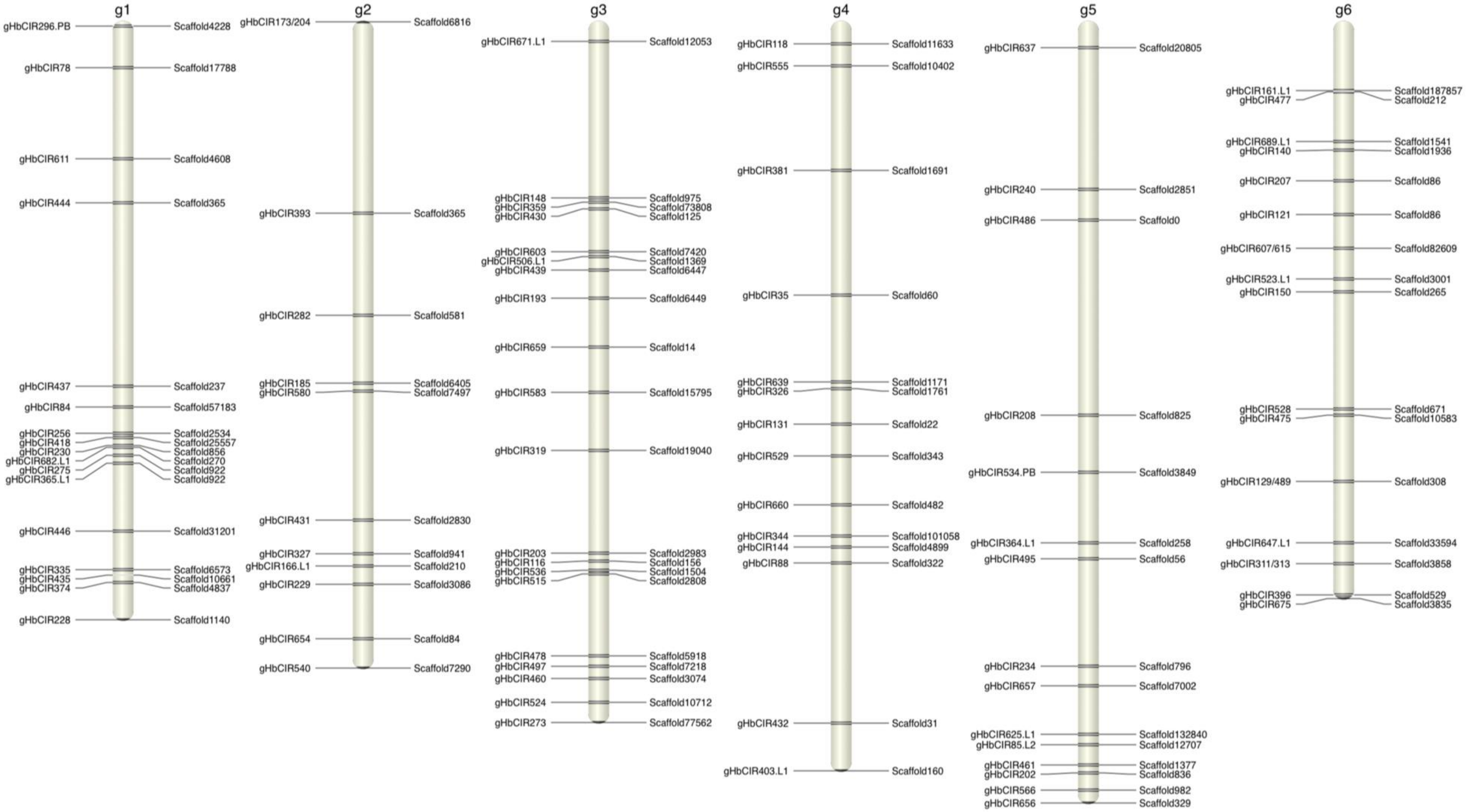

Supplementary Figure 3. Anchoring the genome assembly to the linkage groups of *H. brasiliensis*. 196 RFLP markers from the published genetic map[1] were used for anchoring the scaffolds to the linkage groups. In total, 189 scaffolds that account for 43.6 Mb in length (3% of the assembly size) were anchored.

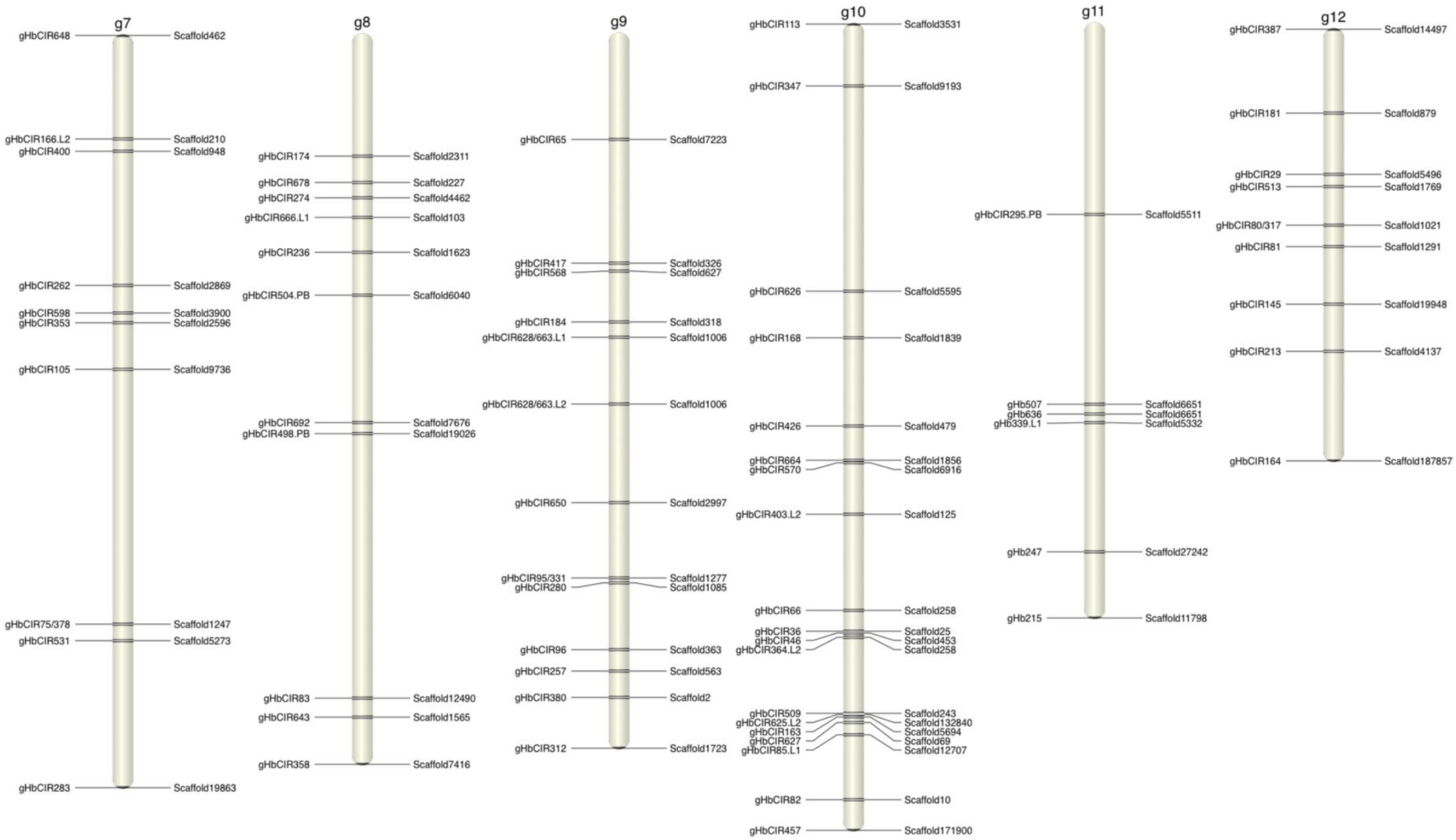

Supplementary Figure 3. Continued.

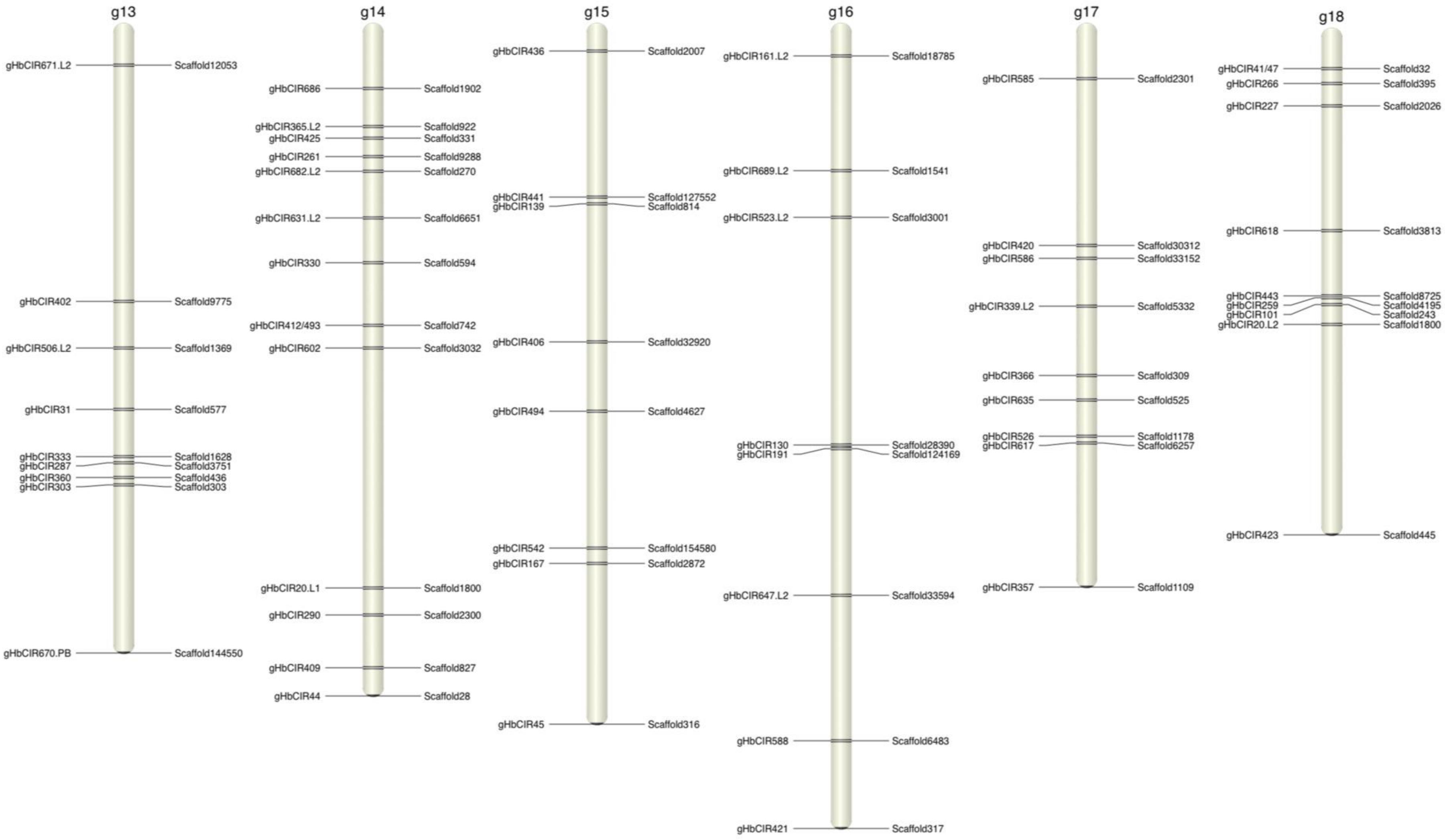

Supplementary Figure 3. Continued.

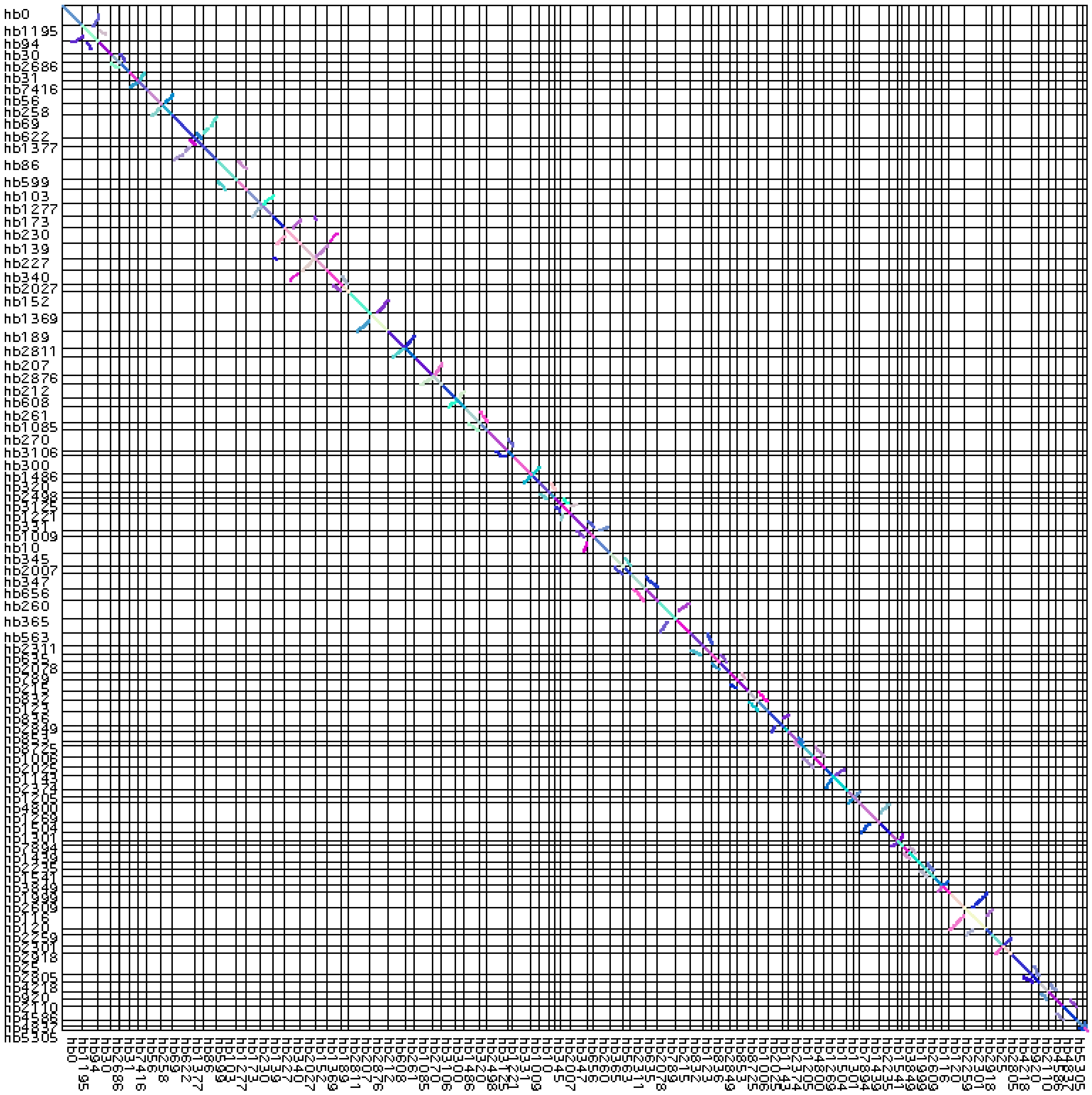

Supplementary Figure 4. BLAST matches between *H. brasiliensis* scaffolds (BLASTP *e*-value cutoff < 10^{-5}). The scaffolds with paralogous gene pairs are shown. Each scaffold shown has more than 15 paralogous genes in another scaffold.

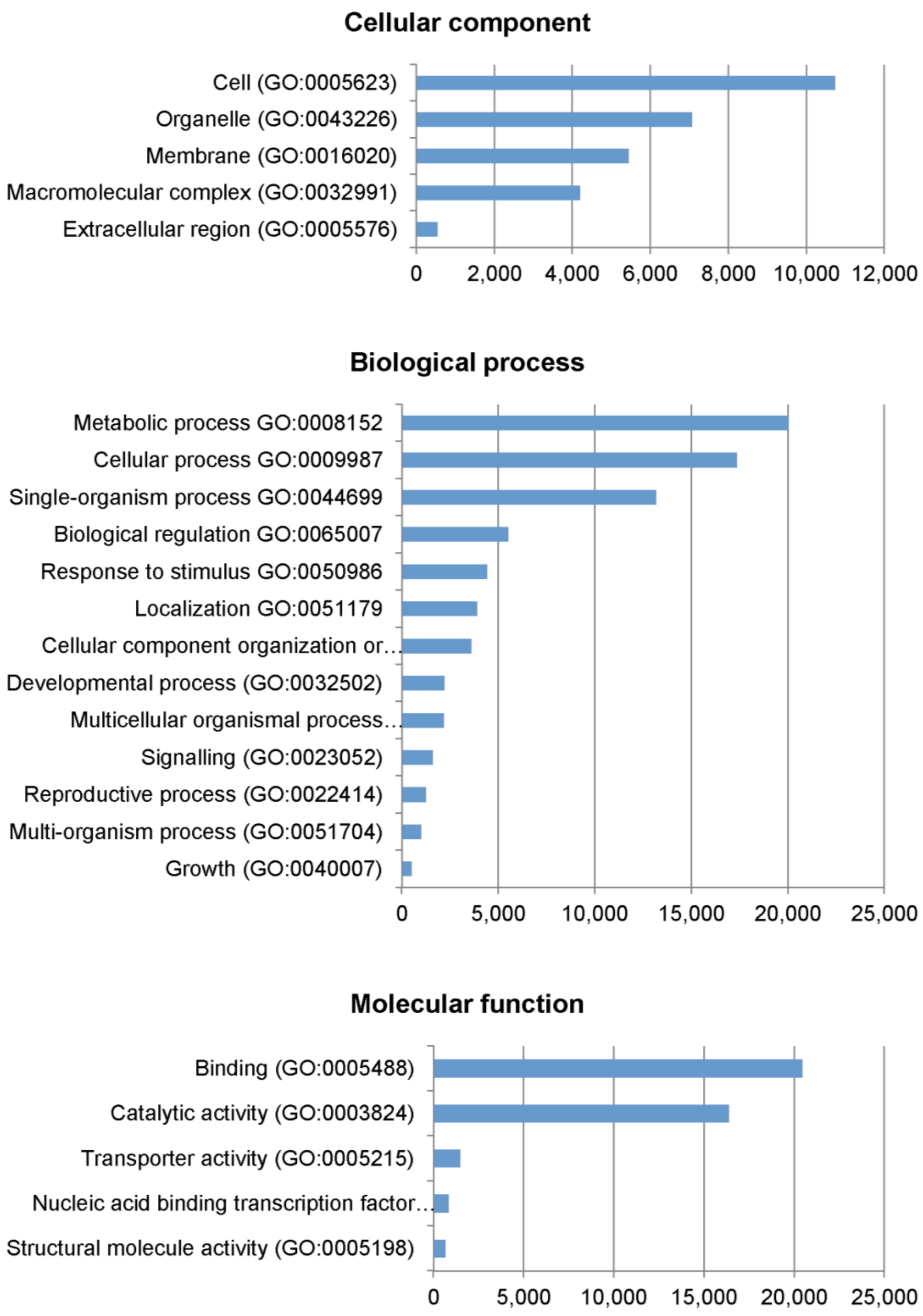

Supplementary Figure 5. Gene ontology classification of *H. brasiliensis*. X-axis represents the number of unique genes with GO annotations.

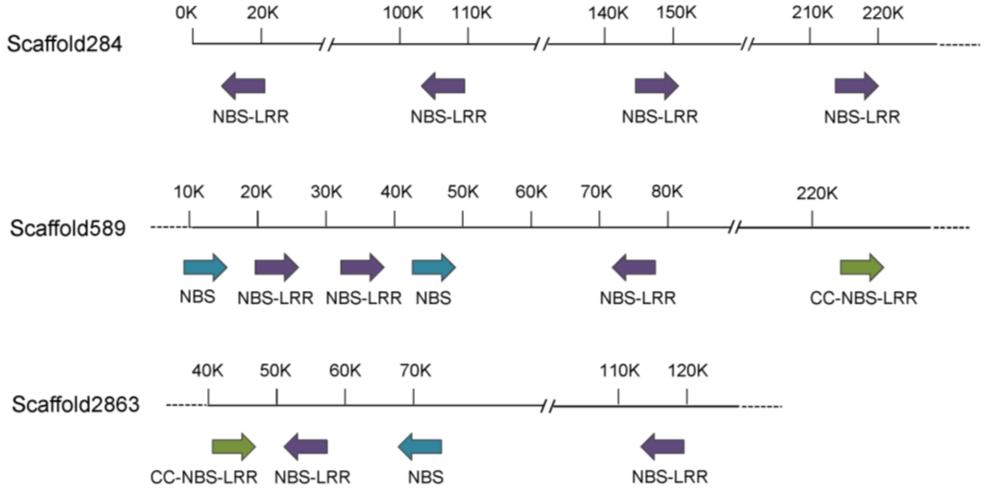

Supplementary Figure 6. NBS gene clusters in the *H. brasiliensis* genome. Some of the NBS resistance genes were found organized in gene clusters along the *H. brasiliensis* genome.

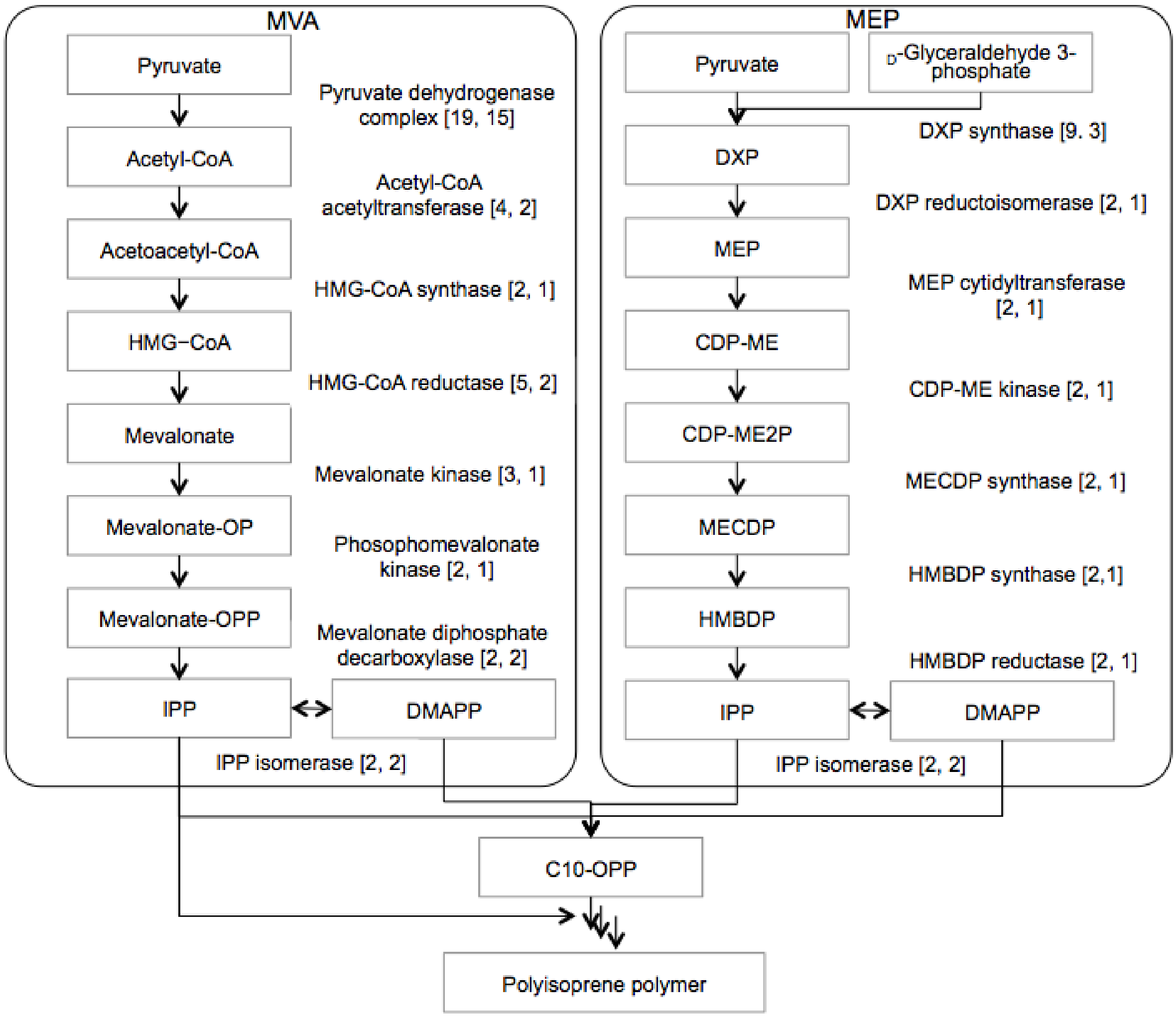

Supplementary Figure 7. Genes involved in rubber biosynthetic pathways in *H. brasiliensis*. The numbers in square brackets represent the numbers of each gene in *H. brasiliensis* and *A. thaliana*, respectively. MVA pathway: HMG-CoA, hydroxymethylglutaryl coenzyme A; Mevalonate-OP, mevalonate monophosphate; Mavalonate-OPP, mevalonate diphosphate; IPP, isopentenyl diphosphate; DMAPP, dimethylallyl diphosphate; C10-OPP, isoprenyl diphosphate. MEP pathway: DXP, 1-deoxy-D-xylulose-5-phosphate; MEP, 2-C-methyl-D-erythritol 4-phosphate; CDP-ME, 2-C-methyl-D-erythritol-4-phosphate; CDP-ME2P, 4-(cytidine 5'-diphospho)-2-C-methyl-D-erythritol; MECDP, 2-C-methyl-D-erythritol 2,4-cyclodiphosphate; HMBDP, 4-hydroxy-3-methyl-but-2-en-1-yl diphosphate.

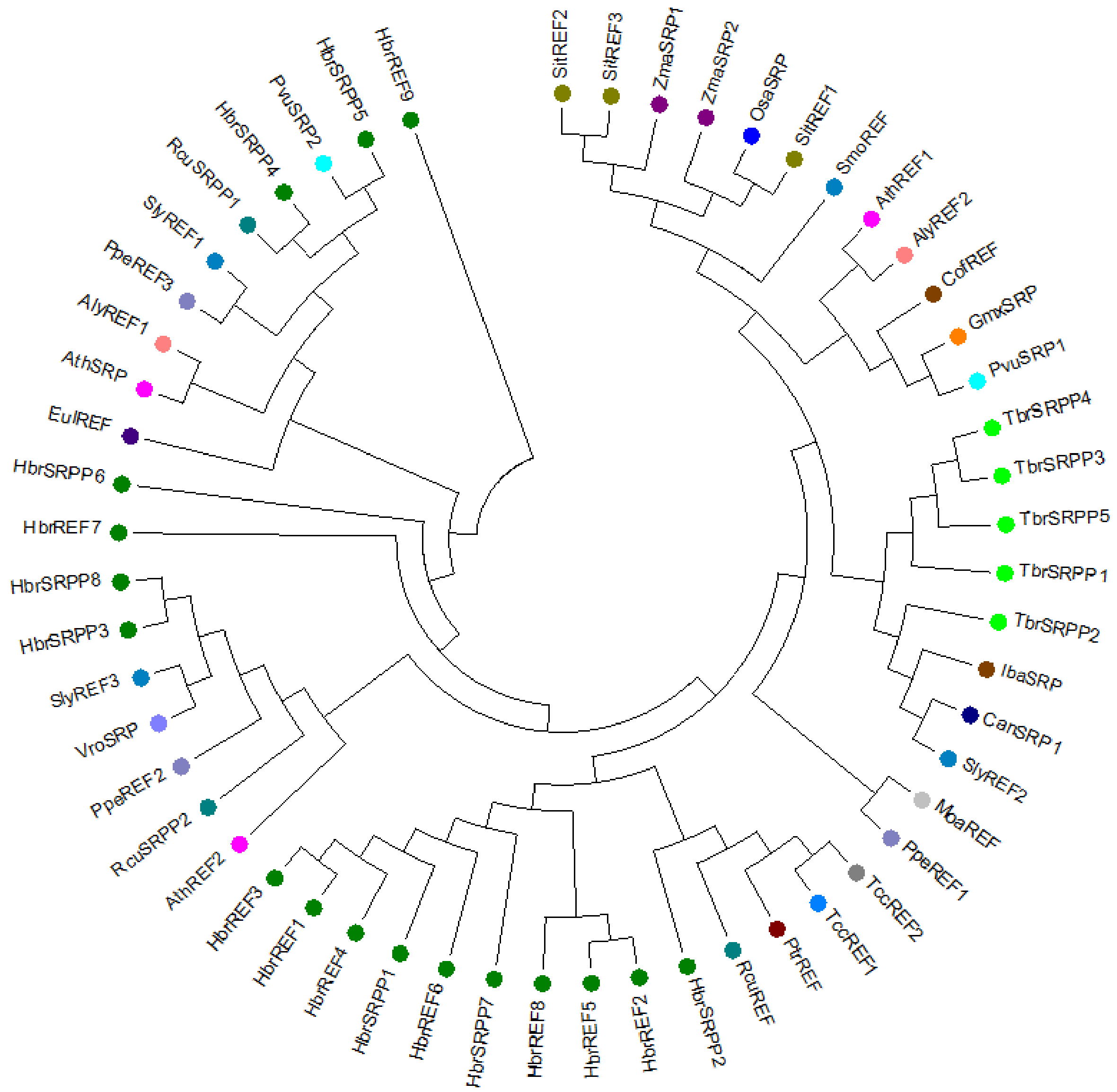

Supplementary Figure 8. Phylogenetic analysis of the REF and SRPP protein family based on the maximum-likelihood method using MEGA (version 6.06). Three letter codes: Ath, *A. thaliana*; Aly, *Arabidopsis lyrata*; Cof, *Copaifera officinalis*; Eul, *Eucommia ulmoides*; Gmx, *Glycine max*; Hbr, *H. brasiliensis*; Iba, *Ipomoea batatas*; Moa, *Morus alba*; Osa, *O. sativa*; Ptc, *Populus trichocarpa*; Pvu, *Phaseolus vulgaris*; Ppe, *Prunus persica*; Rcu, *R. communis*; Sly, *S. lycopersicum*; Sit, *Setaria italica*; Smo, *Selaginella moellendorffii*; Tbr, *Taraxacum brevicorniculatum*; Tcc, *Theobroma cacao*; Vro, *Vitis riparia*; Vvi, *V. vinifera*; Zma, *Zea mays*.

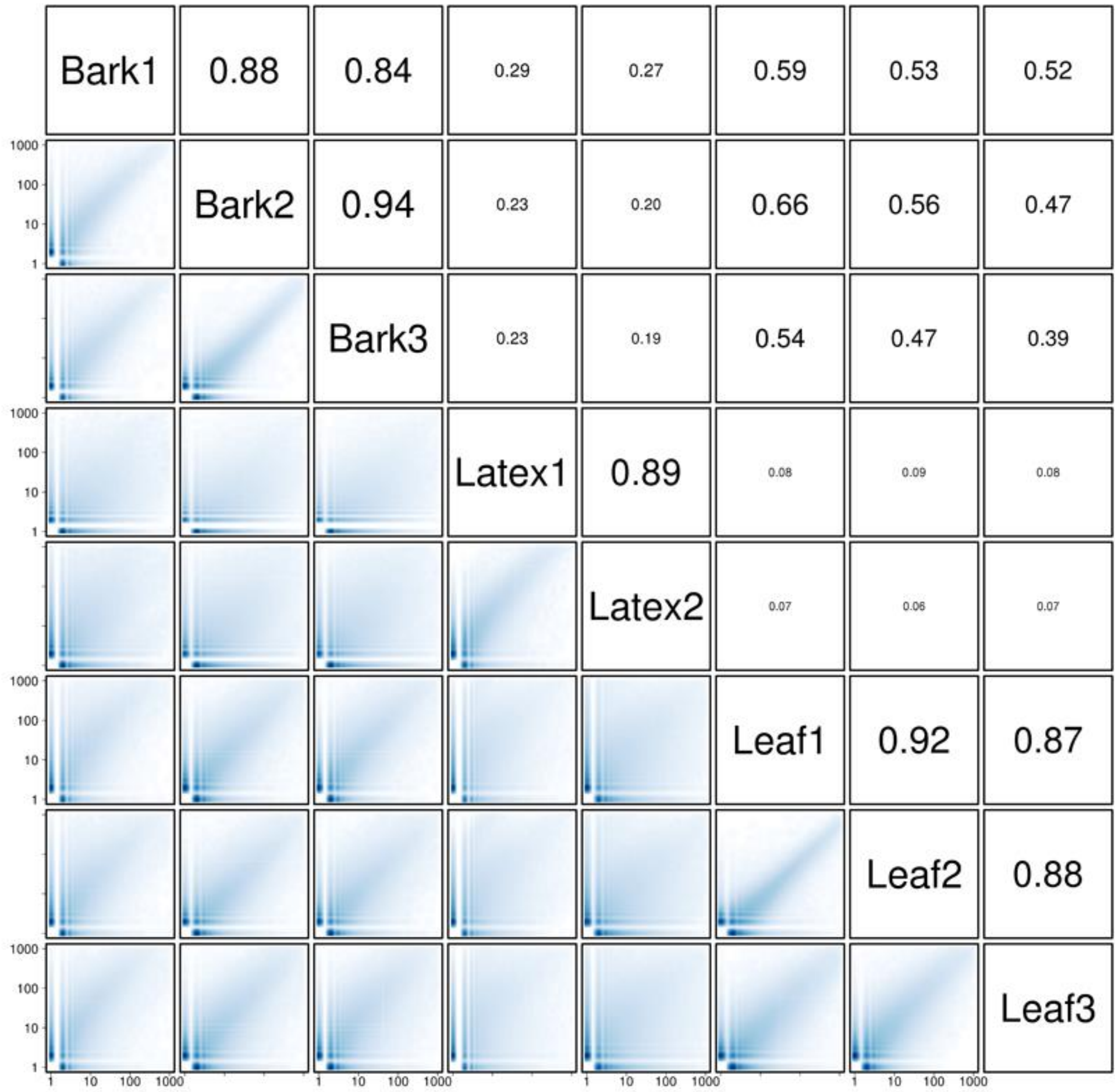

Supplementary Figure 9. Correlation of raw CAGE tag counts per TSS. Pairwise scatter plot of CAGE tag counts per TSS and correlation between all possible pairs of samples. Numbers in the boxes correlate with the values of the correlation coefficients.

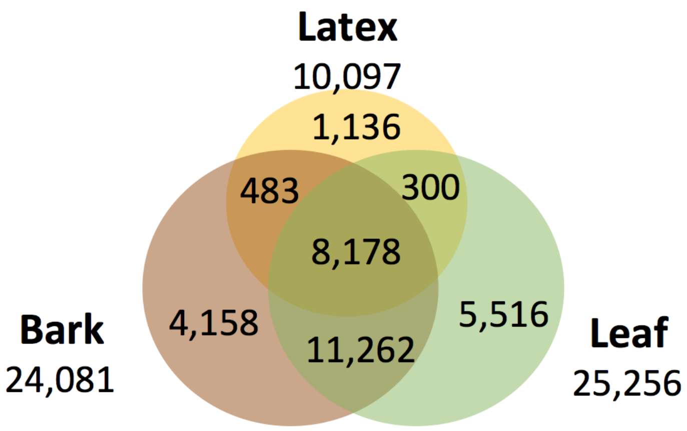

Supplementary Figure 10. Venn diagram of tissue-specific transcription start site clusters. RECLU[2], a reproducible clustering pipeline with multiple scales was used to analyze the CAGE data.

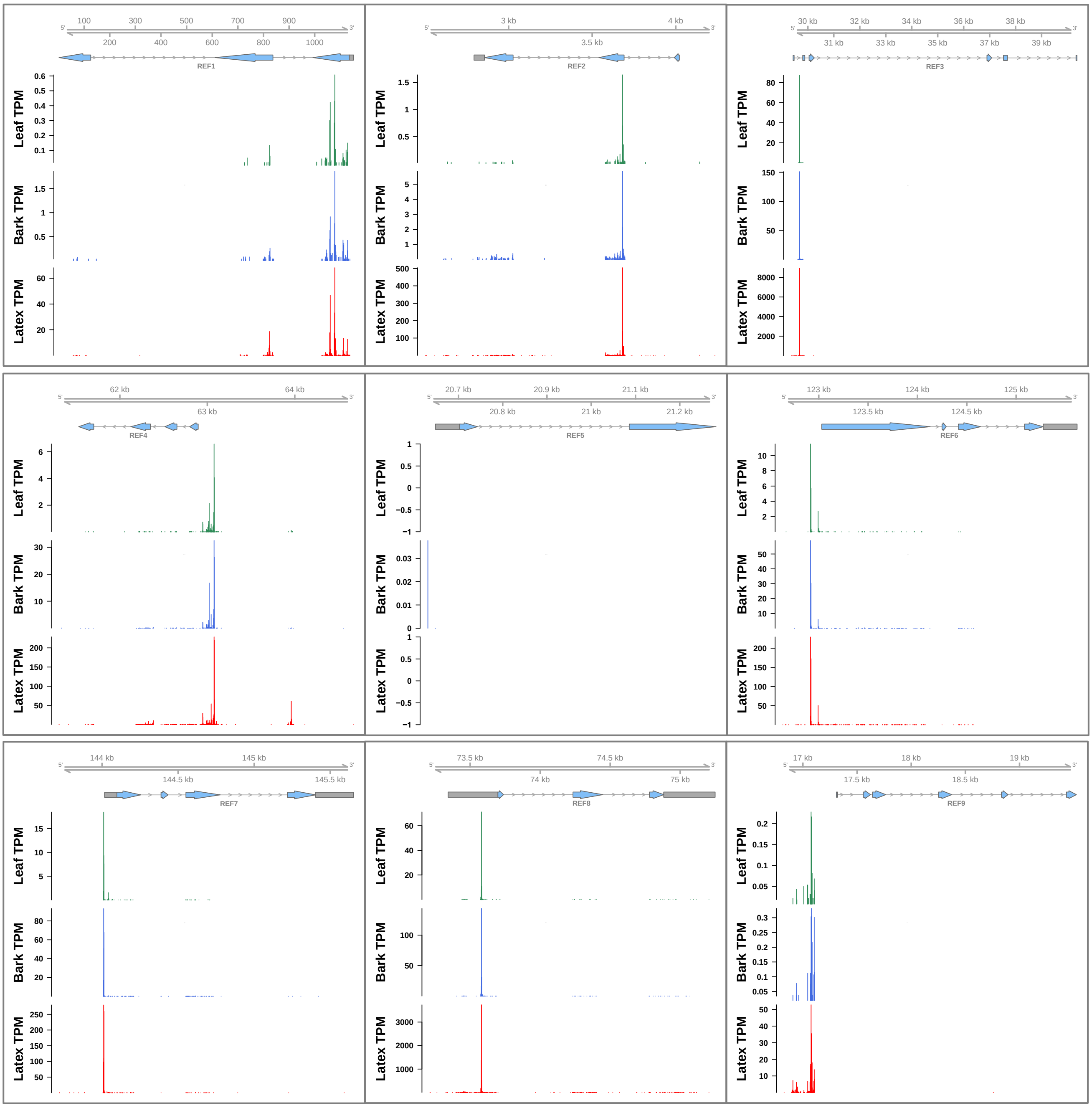

Supplementary Figure 11. CAGE tag cluster distribution for REF1-9 in leaf, bark and latex. The top arrows represent Maker-P predicted gene structures; grey boxes are 5'/3' UTR and blue arrows are CDSs. The histograms show normalized expression (TPM, tags per million) and position of TSSs. Each histogram has a different scale for TPM. The relative locations of each gene in the scaffold are indicated at the top of the figure.

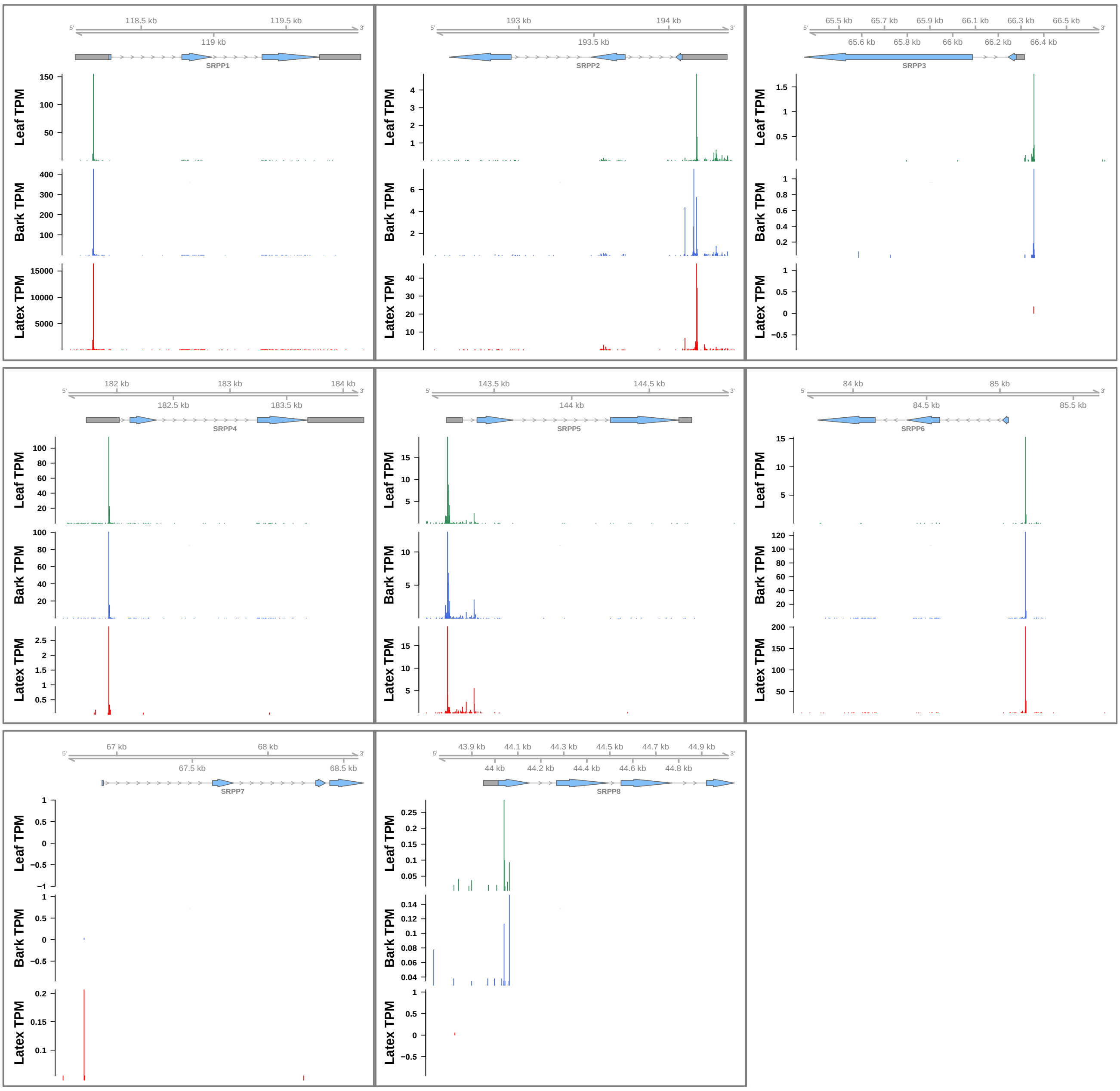

Supplementary Figure 12. CAGE tag cluster distribution for SRPP1-8 in leaf, bark and latex. The top arrows represent Maker-P predicted gene structures; grey boxes are 5'/3' UTR and blue arrows are CDSs. The histograms show normalized expression (TPM) and position of TSSs. Each histogram has a different scale for TPM. The relative locations of each gene in the scaffold are indicated at the top of the figure.

Prenyl-PP biosynthesis

Isopentenyl-diphosphate delta isomerase

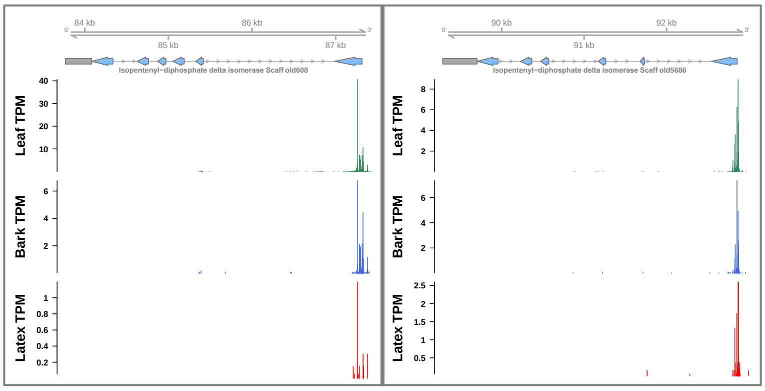

Dimethylallyltransferase

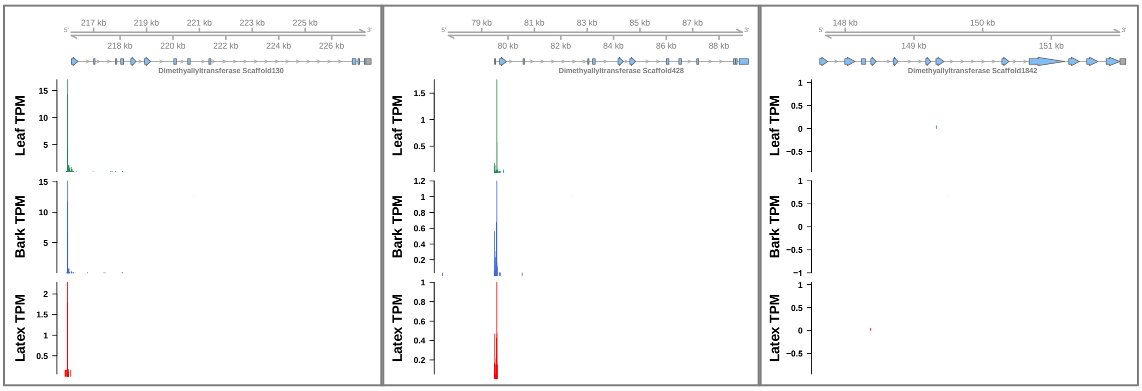

Farnesyl diphosphate synthase

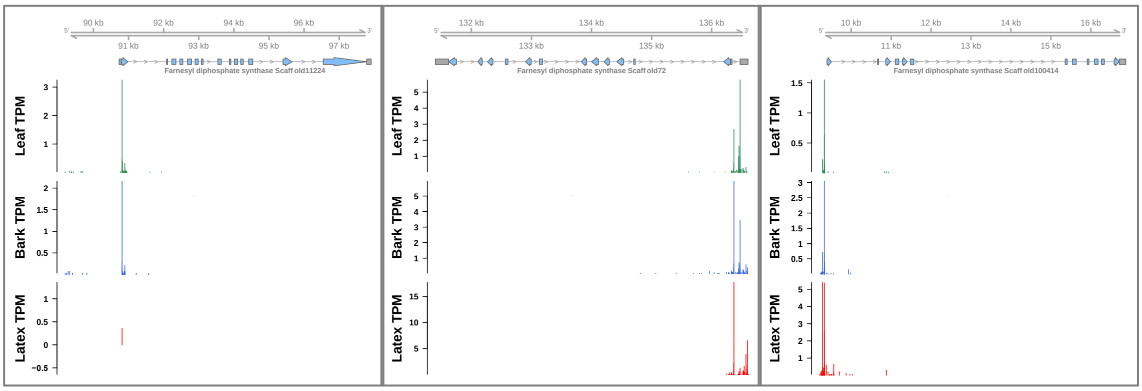

Geranyl diphosphate synthase

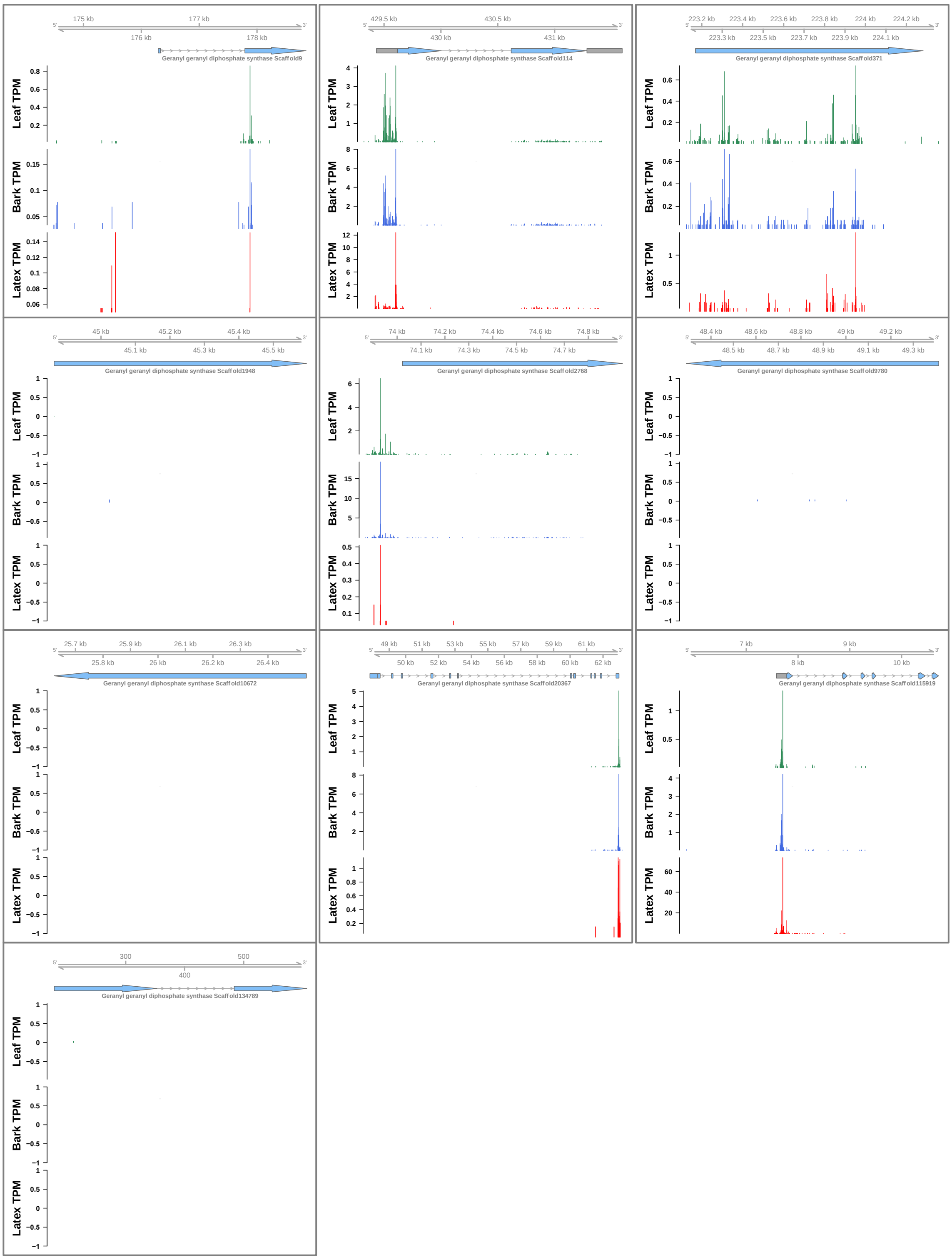

MVA pathway

Pyruvate dehydrogenase

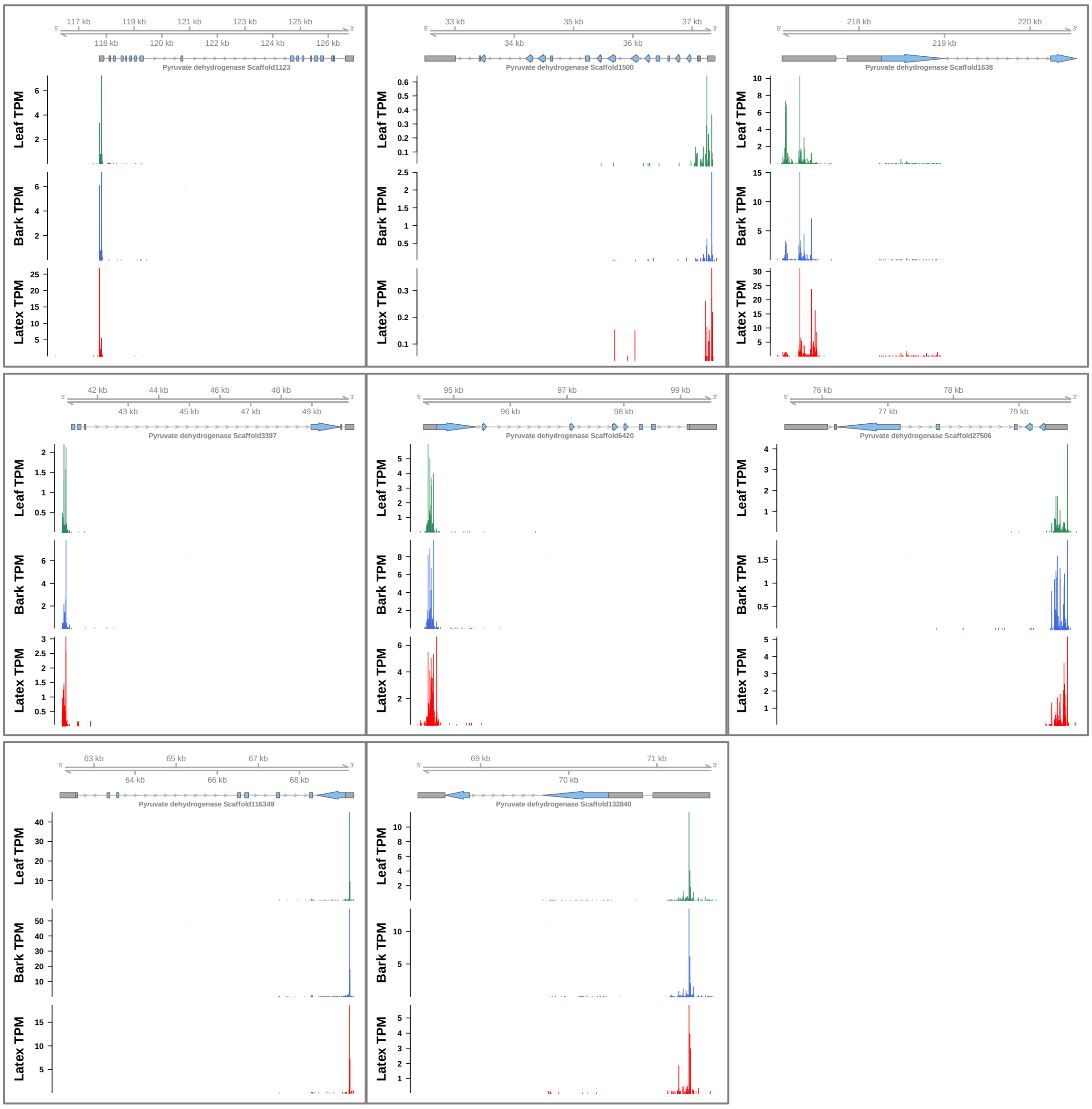

Dihydrolipoyllysine-residue acetyltransferase component of pyruvate dehydrogenase complex

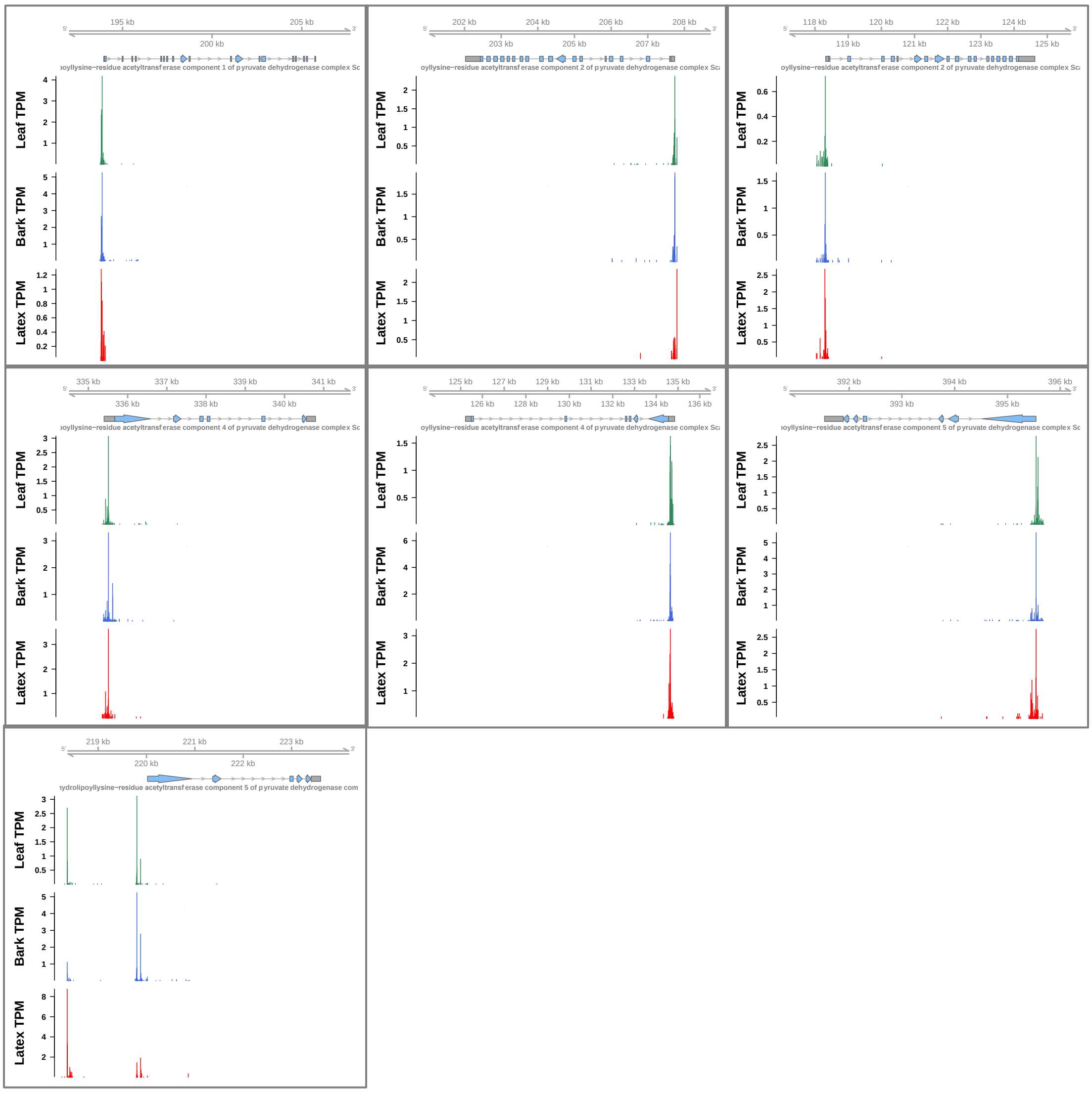

Dihydrolipoamide dehydrogenase

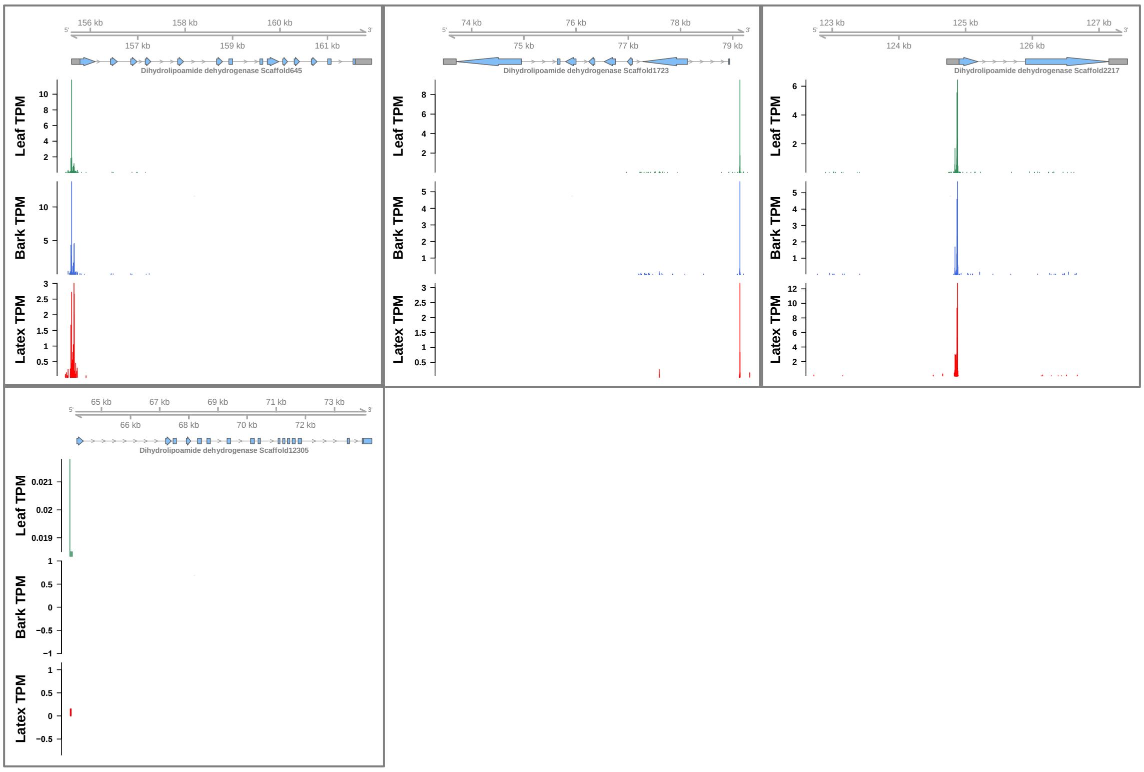

Acetyl-CoA acetyltransferase

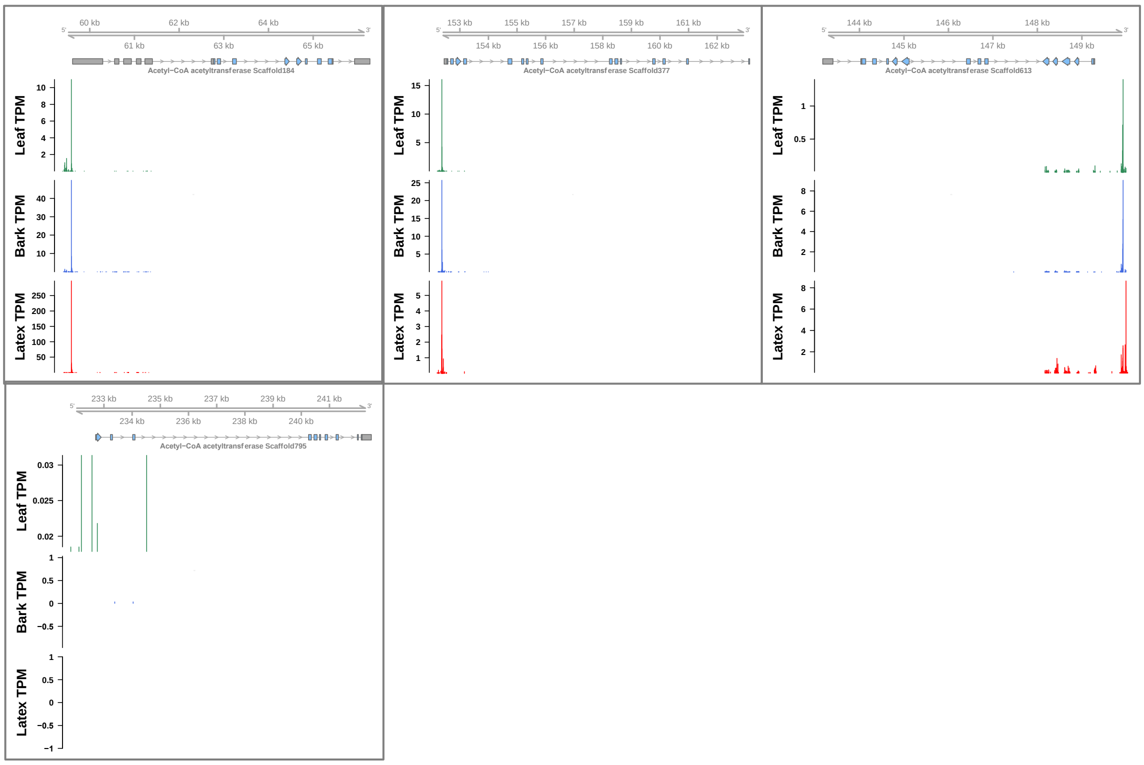

Hydroxymethylglutaryl coenzyme A synthase

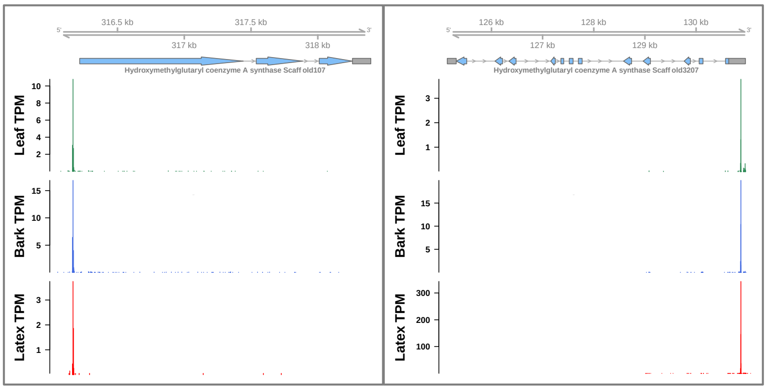

Hydroxymethylglutaryl coenzyme A reductase

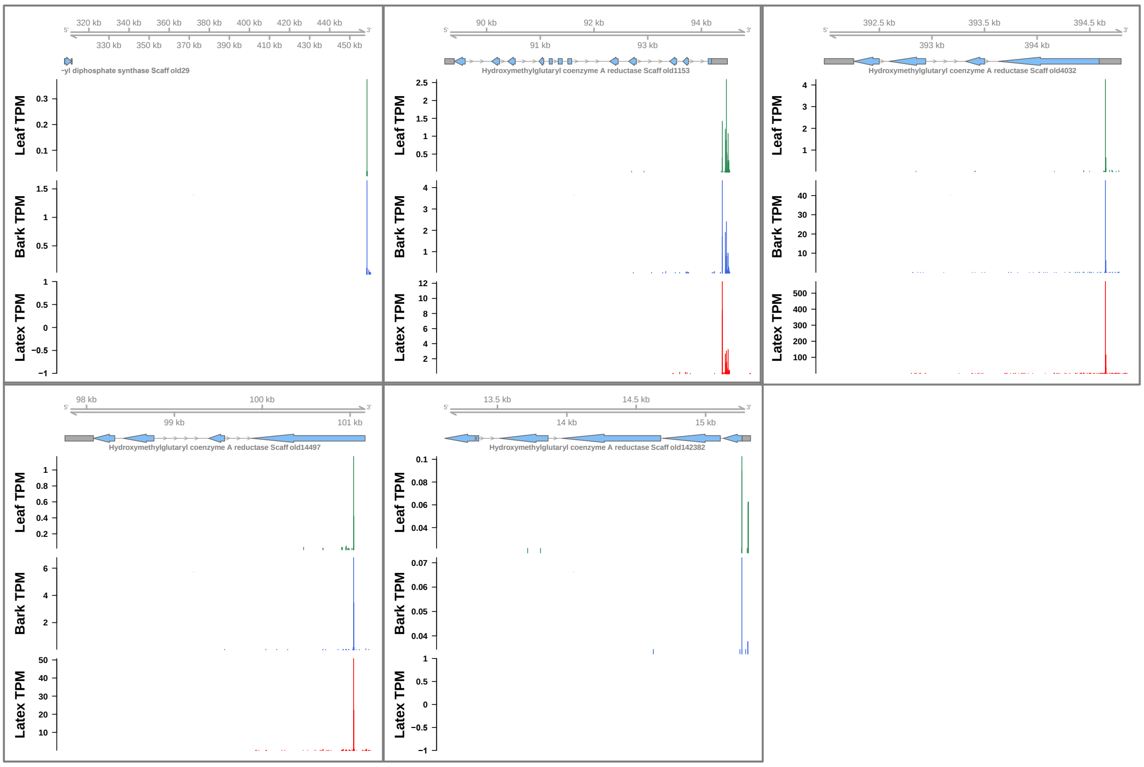

Mevalonate kinase

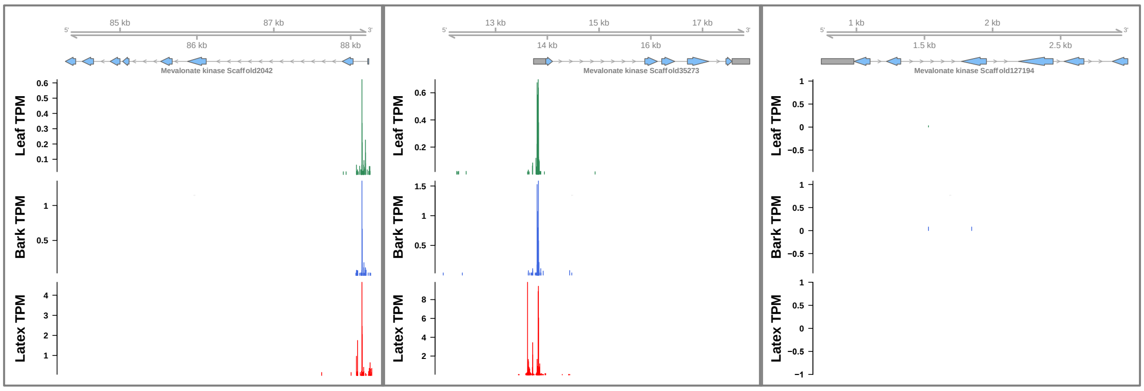

Phosphomevalonate kinase

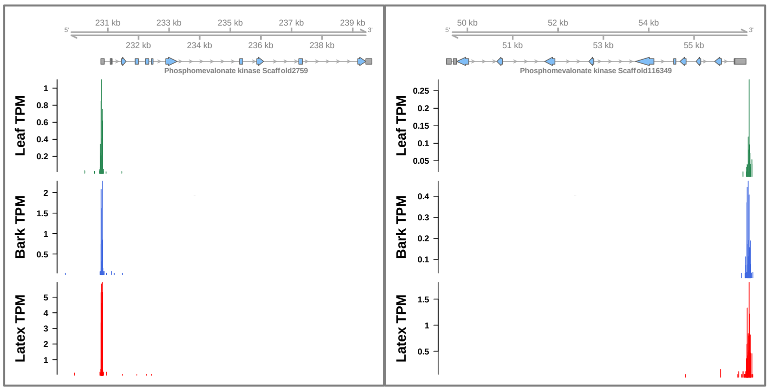

Mevalonate diphosphate decarboxylase

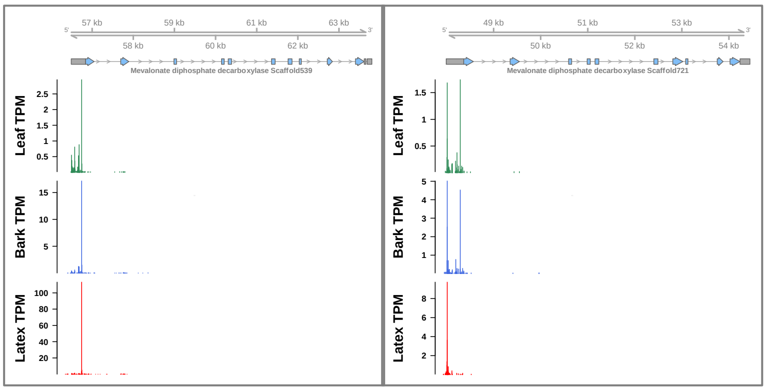

MEP pathway

1-deoxy-D-xylulose-5-phosphate synthase

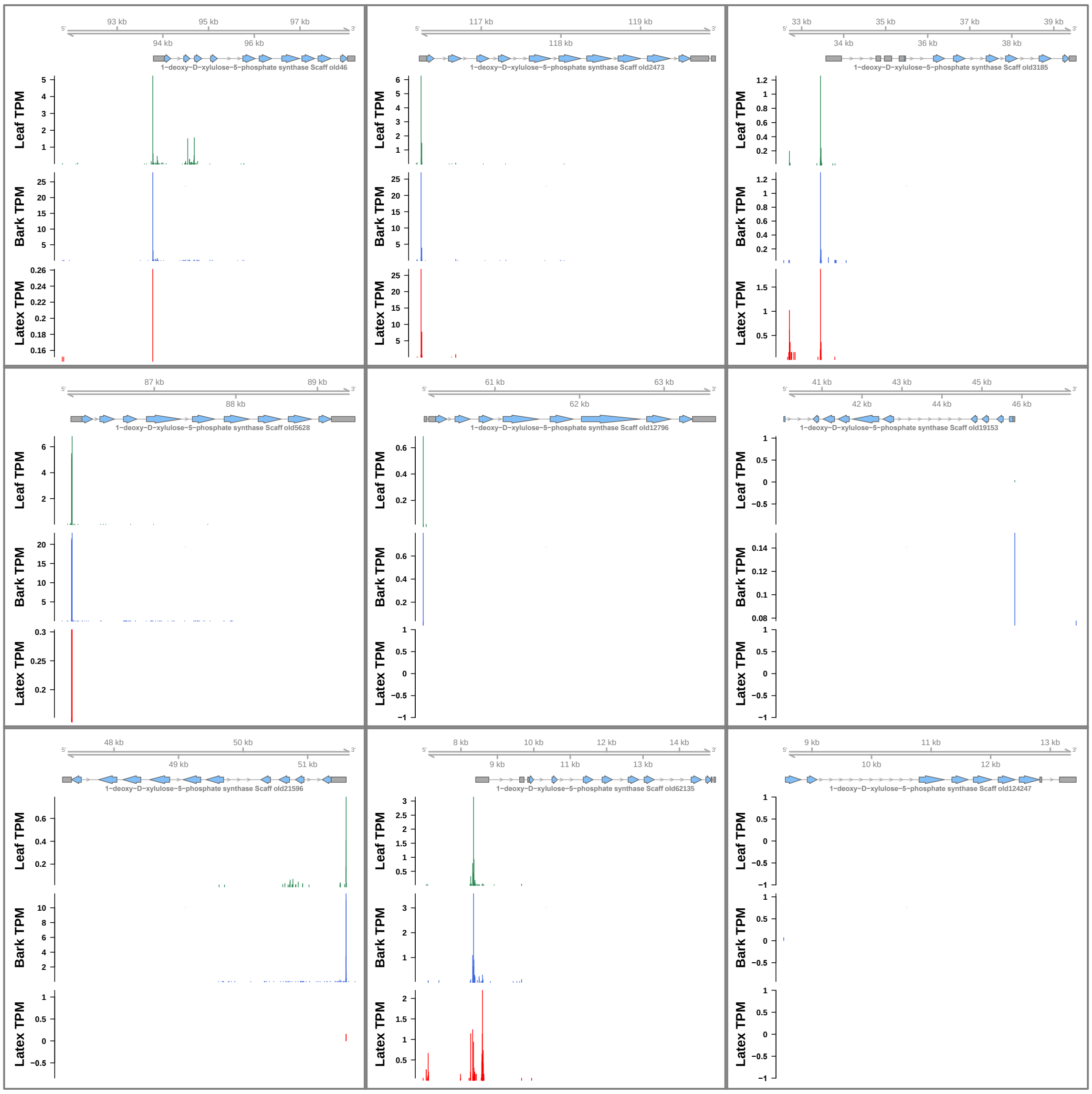

1-deoxy-D-xylulose 5-phosphate reductoisomerase

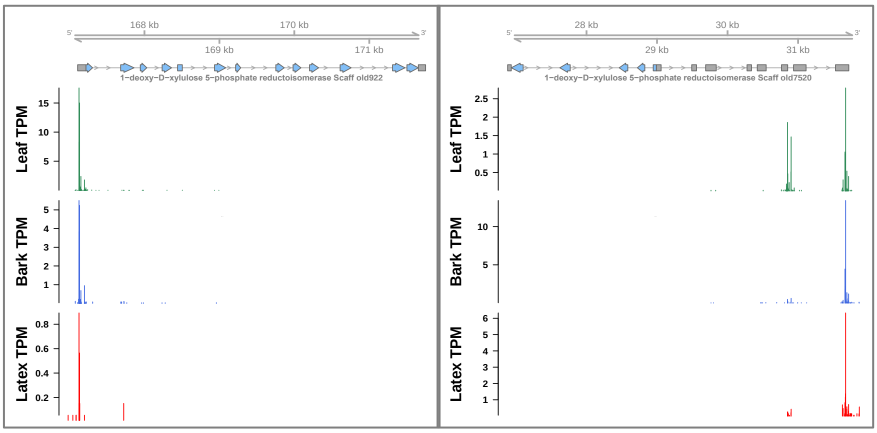

2-C-methyl-D-erythritol 4-phosphate cytidylyltransferase

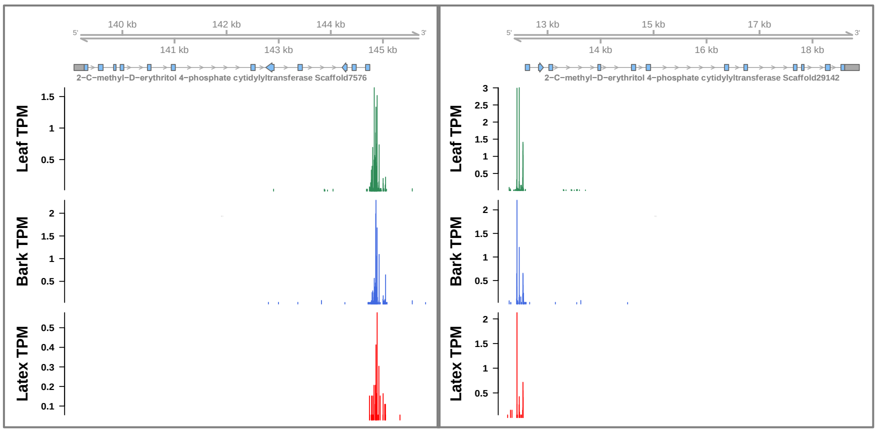

4-(cytidine 5'-diphospho)-2-C-methyl-D-erythritol kinase

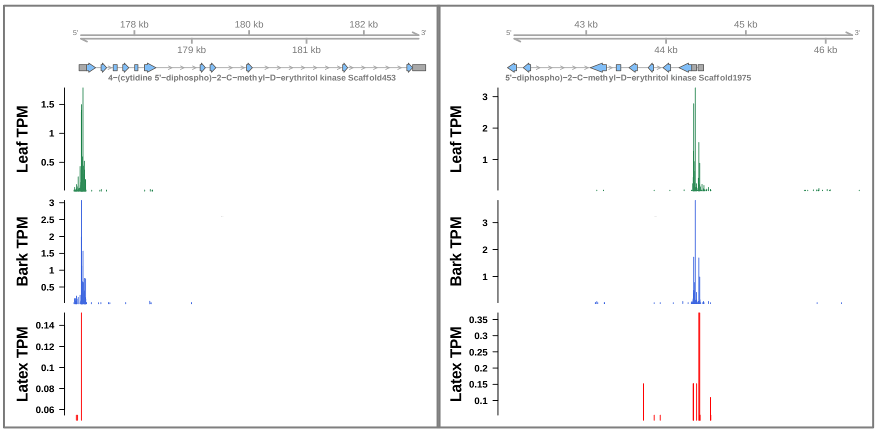

2-C-methyl-D-erythritol 2,4-cyclodiphosphate synthase

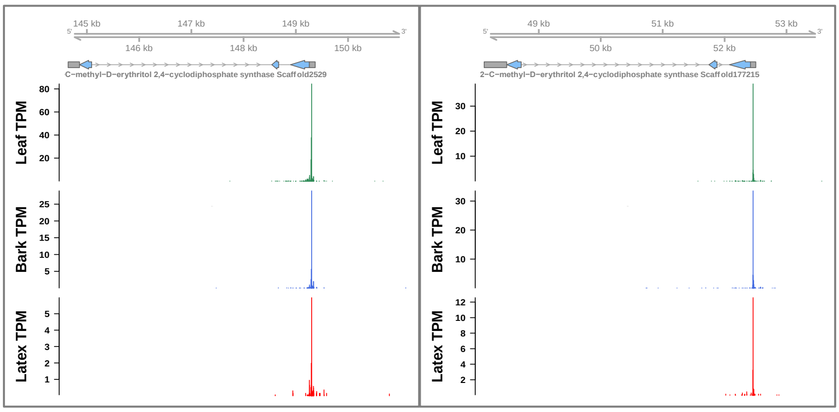

4-hydroxy-3-methylbut-2-en-1-yl diphosphate synthase

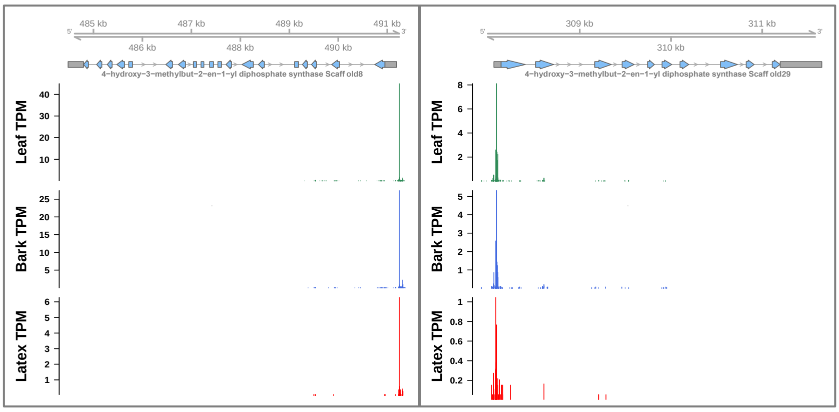

4-hydroxy-3-methylbut-2-enyl diphosphate reductase

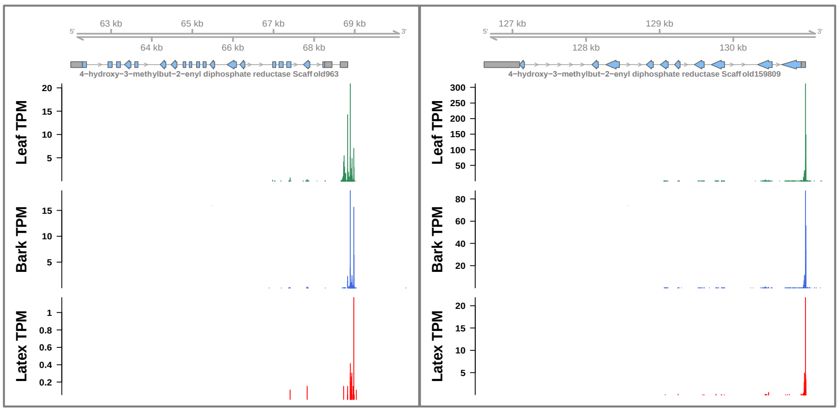

Supplementary Figure 13. CAGE tag cluster distributions for prenyl-PP biosynthetic, MVA and MEP pathway genes in leaf, bark and latex. The top arrows represent Maker-P predicted gene structures; grey boxes are 5'/3' UTR and blue arrows are CDSs. The histograms show normalized expression (TPM) and position of TSSs. Each histogram has a different scale for TPM. The relative locations of each gene in the scaffold are indicated at the top of the figure.

Supplementary Table 1. Summary of the *H. brasiliensis* genome DNA sequencing data.

**Read type**	**Libraries (bp)**	**No. of reads**	**Total size (raw) (Gb)**	**Read length (raw) (bp)**	**Base coverage**
Illumina paired-end	500	617,052,238	93.17	150	43.3
Illumina mate-paired	3,000	402,338,096	40.23	100	18.7
Illumina mate-paired	3,000	21,009,244	3.59	300	1.7
Illumina mate-paired	5,000	420,866,996	42.09	100	19.6
Illumina mate-paired	5,000	33,511,336	6.05	300	2.8
Illumina mate-paired	7,000	476,469,514	47.65	100	22.2
Illumina mate-paired	7,000	12,745,090	2.22	300	1.0
Illumina mate-paired	10,000	538,405,966	53.84	100	25.0
PacBio	10,000	6,603,402	45.25	6,852	21.0

Supplementary Table 2. Reference sequences mapped to the *H. brasiliensis* assembly.

Reference dataset	Number of sequences	Number of sequences mapped	% of sequences mapped	Covered by assembly (%)
EST	51,631	49,998	96.8	94.8
Leaf transcriptome[3]	66,841	66,064	98.8	95.5
Latex transcriptome[3]	48,105	47,618	99.0	94.4
Leaf and latex transcriptome[4]	67,289	66,950	99.5	95.9
Leaf and latex unigene[5]	48,768	45,307	92.9	93.7
Bark transcriptome[3]	91,555	88,493	96.7	96.5
Bark unigene[6]	22,646	20,498	90.5	96.1
Bark transcriptome[7]	28,212	28,065	99.5	94.1

All available *H. brasiliensis* ESTs sequences were downloaded from NCBI and aligned on the assembled scaffolds using BLASTN with a 90% identity cutoff. Transcriptome data from leaf, latex and bark tissues published by Chow *et al.*[3] were first assembled using Oases[8] and subsequently mapped to the scaffolds. Assembled latex, leaf and bark transcripts reported by Rahman *et al.*[4] and Li *et al.*[6] were downloaded from NCBI with accession numbers JT914190-JT981478 and JR344291-JR366936, respectively. Latex and leaf unigenes reported by Xia *et al.*[5] were downloaded from NCBI (accession number GSE26514). Bark transcripts described by Triwitayakorn *et al.*[7] were obtained from http://www4a.biotec.or.th/rubber.

Supplementary Table 3. Organellar sequence insertions in the *H. brasiliensis* genome assembly.

a. Mitochondrial DNA insertions

Range (bp)	Insertion frequency	Insertion size (bp)
<500	297	304,986
500-1,000	65	69,058
1,001-5,000	59	123,355
5,001-10,000	10	45,041
>10,001	14	51,949
**Total**	445	594,389

b. Chloroplast DNA insertions

Range (bp)	Insertion frequency	Insertion size (bp)
<500	338	56,364
500-1,000	27	18,858
1,001-5,000	40	80,567
5,001-10,000	10	63,666
>10,001	1	11,247
**Total**	416	230,702

The *H. brasiliensis* genome assembly was aligned by BLASTN against the *H. brasiliensis* mitochondrial genome (NCBI accession number AP014526) and the chloroplast genome (NCBI accession number HQ285842) to identify organellar sequence insertions into the nuclear genome. Alignments with lengths longer than 50 bp and > 90% identity were retained and categorized by size.

Supplementary Table 4. Repeat composition of *H. brasiliensis* compared with other plant genomes.

	Genome (%)				
**Repeat elements**	***H. brasiliensis***	***R. communis***	***S. bicolor***	***Nicotiana tabacum K326***	***Capsicum annuum***
Retrotransposon	57.3	18.2	54.5	52.1	59.5
SINE	0.0	0.0	0.0	0.3	0.2
LINE	1.1	0.1	0.0	0.3	1.3
LTR/Copia	11.7	4.8	5.2	12.6	6.0
LTR/Gypsy	43.6	11.5	19.0	19.2	51.1
LTR/others	0.9	1.8	30.3	8.6	0.9
DNA transposon	1.2	0.9	7.5	3.3	5.42
Unknown	12.6	31.3	0.1	9.0	14.0

Supplementary Table 5. Number of gene models with homology or functional classification.

	**Database**	**Number**	**Percent (%)**
Annotated	NR	64,587	76.5
	SwissProt	33,552	39.7
	TrEMBL	63,052	74.7
	InterPro	55,312	65.5
	GO	47,790	56.6
	KEGG	61,452	72.8
Unannotated		15,991	18.9
	**Total**	84,440	100

Supplementary Table 6. Non-coding RNAs in the *H. brasiliensis* genome assembly.

ncRNA	Copies (number)	Length occupied (bp)	Genome (%)
Micro RNA	206	26,226	0.0017
Transfer RNA	739	55,362	0.0036
Ribosomal RNA	271	206,670	0.0133
Spliceosomal RNA	192	23,764	0.0015
Small nucleolar RNA	231	25,291	0.0016

Supplementary Table 7. Enriched GO terms in gene clusters specific to *H. brasiliensis*.

Gene Ontology ID	Gene Ontology term	Enrichment FDR
GO:0008270	Zinc ion binding	0.0E0
GO:0043169	Cation binding	0.0E0
GO:0046914	Transition metal ion binding	0.0E0
GO:0046872	Metal ion binding	0.0E0
GO:0003676	Nucleic acid binding	2.4E-257
GO:0005488	Ion binding	2.0E-144
GO:0097159	Binding	4.2E-139
GO:1901363	Organic cyclic compound binding	1.2E-93
GO:0017070	Heterocyclic compound binding	1.2E-93
GO:0030623	U6 snRNA binding	1.8E-6
GO:0004190	U5 snRNA binding	1.1E-4
GO:0070001	Aspartic-type endopeptidase activity	1.1E-4
GO: 0017069	snRNA binding	2.7E-3
GO:0005681	Spliceosomal complex	1.0E-2
GO:0003735	Structural constituents of ribosome	2.1E-2

Supplementary Table 8. Summary of number of family members for each transcription factor in *H. brasiliensis* and selected plant genomes.

Family	*H. brasiliensis*	*A. thaliana*	*S. lycopersicum*	*O. sativa*	*R. communis*
Alfin-like	11	10	10	11	5
AP2	28	18	23	190	11
ARF	22	37	22	48	17
ARID	15	13	14	13	10
ARR-B	16	17	15	22	11
AUX/IAA	44	40	27	62	21
B3	79	78	74	113	40
BBR-BPC	6	17	6	7	5
BES1	13	14	9	6	7
bHLH	158	189	140	181	102
BSD	1	2	1	1	1
bZIP	87	126	68	135	50
C2C2-CO-like	10	18	11	11	4
C2C2-Dof	49	47	33	37	23
C2C2-GATA	41	41	30	35	19
C2C2-LSD	4	12	3	12	4
C2C2-YABBY	14	8	9	15	6
C2H2	173	125	120	167	104
C3H	91	79	62	85	44
CAMTA	8	10	6	7	4
Coactivator p15	4	7	2	3	2
CPP	11	9	4	20	6
CSD	7	4	5	3	5
DBB	3	9	5	5	7
DBP	3	2	3	6	2
DDT	7	6	5	11	4
E2F-DP	10	16	8	10	6
EIL	4	6	9	11	4
ERF	182	151	141	229	100
FAR1	86	26	28	130	20
G2-like	53	61	53	57	32
GeBP	9	23	11	13	4
GNAT	45	44	44	40	45
GRAS	83	37	54	69	48
GRF	16	9	13	19	9
HB	120	118	105	135	67
HMG	21	23	7	14	10
HRT	2	2	1	1	1
HSF	32	25	26	38	18
IWS1	29	12	9	22	7
Jumonji	19	22	20	16	14
LFY	2	1	1	2	1
LIM	21	21	15	16	9
LOB	63	50	47	39	34
LUG	8	9	5	12	4

M-type	63	74	68	38	29
MBF1	6	4	5	4	2
MED6	1	2	2	1	1
MED7	1	4	1	3	1
MIKC	40	72	31	58	10
mTERF	75	40	30	39	35
MYB	202	160	139	124	61
MYB-related	101	99	75	91	94
NAC	179	137	101	170	95
NF-X1	3	1	1	2	–
NF-YA	17	21	10	25	6
NF-YB	23	27	27	16	14
NF-YC	18	21	16	19	11
NOZZLE	3	1	1	–	–
OFP	23	17	24	31	16
Orphans	85	99	72	91	114
PHD	57	56	50	52	40
PLATZ	19	15	21	21	11
Pseudo ARR-B	7	7	5	14	6
RAV	7	7	3	4	4
RB	3	2	1	4	2
Rcd1-like	3	3	13	5	3
RWP-RK	15	17	10	15	10
S1Fa-like	2	4	1	2	1
SAP	2	1	2	–	1
SBP	29	30	17	29	15
SET	53	55	48	46	33
SNF2	42	59	62	54	34
SOH1	2	2	1	3	–
SRS	12	16	9	6	5
STAT	2	4	1	1	1
SWI/SNF-BAF60b	20	19	15	11	11
SWI/SNF-SWI3	4	4	6	4	6
TAZ	7	11	7	12	3
TCP	35	33	36	23	22
Tify	24	27	17	23	11
TRAF	31	30	18	62	13
Trihelix	52	31	25	36	27
TUB	15	19	11	28	7
ULT	4	3	3	2	2
VOZ	3	3	2	2	1
Whirly	3	4	2	2	2
WRKY	112	90	81	128	58
zf-HD	27	18	22	15	11
**Total**	3,142	2,942	2,396	3,100	1,776

Supplementary Table 9. Summary of number of disease resistance genes in *H. brasiliensis* and selected Euphorbiaceae genomes.

**Type**	***H. brasiliensis***	***R. communis***	***M. esculenta***	***J. curcas***
TIR-NBS	4	7	3	15
TIR-NBS-LRR	15	25	27	40
CC-NBS	35	17	15	6
CC-NBS-LRR	45	21	40	19
NBS-LRR	186	67	124	91
NBS	198	95	103	104
**Total**	483	232	312	275

Supplementary Table 10. Jasmonic acid, ethylene and salicylic acid receptor genes identified in *H. brasiliensis*.

Gene name	Description	Scaffold	Start	End
COI1	Coronatine insensitive 1	Scaffold5054	125,489	121,894
		Scaffold12564	63,741	68,657
ETR1	Ethylene receptor 1	Scaffold949	142,689	130,105
		Scaffold4627	71,355	63,041
		Scaffold853	81,746	78,784
		Scaffold4375	95,628	82,405
NPR1	Nonexpresser of PR genes 1	Scaffold322	157,862	161,990
		Scaffold5000	299,616	294,625
NPR3	Nonexpresser of PR genes 3	Scaffold1409	143,759	139,510
		Scaffold5499	13,134	7,635
NPR4	Nonexpresser of PR genes 4	Scaffold5203	33,116	2,5364

Supplementary Table 11. Isoprenoid and rubber biosynthesis pathway genes in *H. brasiliensis*.

a. Prenyl-PP biosynthesis

Gene name	EC No.	Scaffold	Start	End
Isopentenyl-diphosphate delta isomerase	5.3.3.2	Scaffold608	87,313	84,083
		Scaffold5686	92,855	89,702
Dimethyallyltransferase	2.5.1.1	Scaffold130	216,160	227,291
		Scaffold428	79,505	88,623
		Scaffold1842	147,630	151,997
Farnesyl diphosphate synthase	2.5.1.10	Scaffold72	136,310	131,621
		Scaffold11224	90,794	97,780
		Scaffold100414	9,395	16,712
Geranyl geranyl diphosphate synthase	2.5.1.29	Scaffold9	176,292	178,848
		Scaffold114	429,619	431,283
		Scaffold371	223,169	224,281
		Scaffold1948	44,864	45,595
		Scaffold2768	74,022	74,942
		Scaffold9780	49,412	48,291
		Scaffold10672	26,539	25,622
		Scaffold20367	62,971	48,270
		Scaffold115919	7,773	10,714
		Scaffold134789	179	606

b. MVA pathway

Gene name	EC No.	Scaffold	Start	End
Pyruvate dehydrogenase	1.2.4.1	Scaffold1123	118,126	126,191
		Scaffold1500	37,111	33,434
		Scaffold1638	218,260	220,542
		Scaffold3397	41,153	49,943
		Scaffold6420	94,702	99,160
		Scaffold27506	79,406	76,217

		Scaffold116349	69,117	62,554
		Scaffold132840	70,448	68,595
Dihydrolipoyllysine-residue acetyltransferase component 1 of pyruvate dehydrogenase complex	2.3.1.12	Scaffold800	193,988	205,764
Dihydrolipoyllysine-residue acetyltransferase component 2 of pyruvate dehydrogenase complex		Scaffold2477	207,634	202,430
		Scaffold5306	118,413	124,137
Dihydrolipoyllysine-residue acetyltransferase component 4 of pyruvate dehydrogenase complex		Scaffold110	335,675	340,558
		Scaffold3411	134,555	125,489
Dihydrolipoyllysine-residue acetyltransferase component 5 of pyruvate dehydrogenase complex		Scaffold207	395,540	391,889
		Scaffold2876	220,022	223,402
Dihydrolipoamide dehydrogenase	1.8.1.4	Scaffold645	155,781	161,585
		Scaffold1723	78,933	73,703
		Scaffold2217	124,902	127,146
		Scaffold12305	64,155	73,997
Acetyl-CoA acetyltransferase	2.3.1.9	Scaffold184	62,774	65,426
		Scaffold377	152,548	163,126
		Scaffold613	149,270	144,049
		Scaffold795	232,731	242,156
Hydroxymethylglutaryl coenzyme A synthase	2.3.3.10	Scaffold3207	130,638	125311
		Scaffold107	316,217	318259
Hydroxymethylglutaryl coenzyme A reductase	1.1.1.34	Scaffold116	458,599	461,395
		Scaffold1153	94,187	89,398
		Scaffold4032	394,588	392,257
		Scaffold14497	101,170	98,081
		Scaffold142382	15,127	13,340
Mevalonate kinase	2.7.1.36	Scaffold2042	88,233	84,287
		Scaffold35273	13,971	17,568
		Scaffold127194	2,993	981
Phosphomevalonate kinase	2.7.4.2	Scaffold2759	231,117	239,427

		Scaffold116349	55,899	49,762
Mevalonate diphosphate decarboxylase	4.1.1.33	Scaffold539	56,841	63,613
		Scaffold721	48,364	54,235

c. MEP pathway

**Gene name**	**EC No.**	**Scaffold**	**Start**	**End**
1-deoxy-D-xylulose-5-phosphate synthase	2.2.1.7	Scaffold46	94,033	98,039
		Scaffold2473	116,318	119,628
		Scaffold3185	35,444	39,363
		Scaffold5628	86,114	89,169
		Scaffold12796	60,301	63,332
		Scaffold19153	45,776	40,045
		Scaffold21596	51,361	47,341
		Scaffold62135	9,879	14,871
		Scaffold124247	8,553	12,822
1-deoxy-D-xylulose 5-phosphate reductoisomerase	1.1.1.267	Scaffold922	167,219	171,654
		Scaffold7520	28,990	26,935
2-C-methyl-D-erythritol 4-phosphate cytidylyltransferase	2.7.7.60	Scaffold7576	144,747	139,268
		Scaffold29142	12,579	18,607
4-(cytidine 5'-diphospho)-2-C-methyl-D-erythritol kinase	2.7.1.148	Scaffold453	177,164	182,850
		Scaffold1975	44,319	42,012
2-C-methyl-D-erythritol 2,4-cyclodiphosphate synthase	4.6.1.12	Scaffold2529	149,258	144,857
		Scaffold177215	52,417	48,481
4-hydroxy-3-methylbut-2-en-1-yl diphosphate synthase	1.17.4.3	Scaffold8	490,950	484,797
		Scaffold29	308,139	311,196
4-hydroxy-3-methylbut-2-enyl diphosphate reductase	1.17.1.2	Scaffold963	68,257	62,290
		Scaffold159809	130,924	127,098

d. Rubber polymerization

Gene	Description	Scaffold	Start	End
CPT1	*cis*-Prenyltransferase	Scaffold548	63,519	64,387
CPT2		Scaffold6070	25,320	24,430
CPT3		Scaffold313	92,897	92,007
CPT4		Scaffold313	138,258	137,156
CPT5		Scaffold548	45,869	46,662
CPT6		Scaffold120	677,195	678,316
CPT7		Scaffold1999	103,613	105,321
CPTL	*cis*-Prenyltransferase-like	Scaffold4659	115,978	112,945
SRPP1	Small rubber particle protein	Scaffold1741	118,276	119,728
SRPP2		Scaffold617	194,088	192,536
SRPP3		Scaffold24714	66,278	65,348
SRPP4		Scaffold2078	182,116	183,686
SRPP5		Scaffold635	143,390	144,689
SRPP6		Scaffold345	85,056	83,759
SRPP7		Scaffold1741	66,904	68,630
SRPP8		Scaffold2016	44,014	45,040
REF1	Rubber elongation factor	Scaffold111648	1,133	611
REF2		Scaffold149683	4,020	2,855
REF3		Scaffold1741	29,448	40,351
REF4		Scaffold345	62,894	61,530
REF5		Scaffold152868	20,703	21,280
REF6		Scaffold1741	124,249	125,273
REF7		Scaffold1741	144,098	145,403
REF8		Scaffold1741	73,698	74,881
REF9		Scaffold1741	17,312	19,523

Supplementary Table 12. CAGE read mapping statistics.

Sample	Cleaned reads	Mapped reads	Mapped reads (MapQ >= 99%)	No. of TSS positions
Bark 1	34,133,792	8,533,449	4,297,529	708,395
Bark 2	67,555,612	16,888,904	9,669,744	1,305,412
Bark 3	68,164,388	17,041,098	8,851,884	1,230,638
Latex 1	20,392,552	5,098,139	3,292,995	465,411
Latex 2	58,093,928	14,523,483	9,150,459	778,828
Leaf 1	97,993,704	24,498,427	18,016,471	1,397,408
Leaf 2	57,648,544	14,412,137	10,629,899	1,065,226
Leaf 3	79,898,692	19,974,674	15,285,261	1,270,964

Raw sequences were cleaned and adapter-trimmed using the TagDust2[9] program. The clean reads were mapped with BWA[10] to the *H. brasiliensis* genome and reads with mapping quality >= 99% were retained for further analysis**.**

Supplementary Table 13. Location of CAGE transcription start site clusters (TSCs).

Region category	No. of TSCs
Upstream < 2,000 bp	10,786
Inside gene	17,030
Downstream < 500 bp	354
Intergenic region	3,552

TSCs were defined using RECLU[2], a pipeline that is used to discover reproducible TSSs and their alternative regulation from CAGE data.

Supplementary Table 14. Enriched GO terms in latex-specific genes.

Gene Ontology ID	Gene Ontology term	Enrichment FDR
GO:0005623	Cell	5.03E-06
GO:0044464	Cell part	5.03E-06
GO:0005622	Intracellular	5.03E-06
GO:0044424	Intracellular part	6.39E-06
GO:0043229	Intracellular organelle	2.87E-04
GO:0043226	Organelle	2.87E-04
GO:0003677	DNA binding	6.64E-04
GO:0043231	Intracellular membrane-bounded organelle	1.36E-03
GO:0043227	Membrane-bounded organelle	1.36E-03
GO:0065007	Biological regulation	1.80E-03
GO:0009058	Biosynthetic process	9.37E-03
GO:0044699	Single-organism process	9.37E-03
GO:0050789	Regulation of biological process	1.08E-02
GO:0044249	Cellular biosynthetic process	1.11E-02
GO:0005634	Nucleus	1.32E-02
GO:0010817	Regulation of hormone levels	2.11E-02
GO:1901576	Organic substance biosynthetic process	2.29E-02
GO:0012505	Endomembrane system	4.97E-02
GO:0031177	Phosphopantetheine binding	4.97E-02
GO:0009889	Regulation of biosynthetic process	4.97E-02

Supplementary Table 15. Enriched GO terms in bark-specific genes.

Gene Ontology ID	Gene Ontology term	Enrichment FDR
GO:0003700	Transcription factor activity, sequence-specific DNA binding	2.05E-12
GO:0006355	Regulation of transcription, DNA-templated	4.86E-12
GO:0005667	Transcription factor complex	1.69E-11
GO:0005524	ATP binding	2.39E-10
GO:0003824	Catalytic activity	1.47E-07
GO:0005886	Plasma membrane	4.38E-07
GO:0005634	Nucleus	1.68E-06
GO:0016021	Integral component of membrane	1.15E-05
GO:0010075	Regulation of meristem growth	3.94E-05
GO:0043565	Sequence-specific DNA binding	5.43E-05
GO:1901700	Response to oxygen-containing compound	1.40E-04
GO:0071555	Cell wall organization	1.97E-04
GO:0009505	Plant-type cell wall	2.08E-04
GO:0009855	Determination of bilateral symmetry	3.08E-04
GO:0010089	Xylem development	3.62E-04
GO:0003682	Chromatin binding	4.36E-04
GO:0044765	Single-organism transport	7.45E-04
GO:0047262	Polygalacturonate 4-alpha-galacturonosyltransferase activity	8.76E-04
GO:0006950	Response to stress	1.08E-03
GO:0009944	Polarity specification of adaxial/ abaxial axis	1.10E-03
GO:0005215	Transporter activity	1.10E-03
GO:0045489	Pectin biosynthetic process	1.19E-03
GO:0009416	Response to light stimulus	1.19E-03
GO:0009506	Plasmodesma	1.19E-03
GO:0005985	Sucrose metabolic process	1.25E-03
GO:0048439	Flower morphogenesis	1.48E-03
GO:0048519	Negative regulation of biological process	1.48E-03
GO:0006468	Protein phosphorylation	1.49E-03
GO:0046983	Protein dimerization activity	1.50E-03

GO:0042623	ATPase activity, coupled	1.57E-03
GO:0010014	Meristem initiation	1.66E-03
GO:0016051	Carbohydrate biosynthetic process	1.82E-03
GO:0005976	Polysaccharide metabolic process	2.99E-03
GO:0048608	Reproductive structure development	3.47E-03
GO:0000785	Chromatin	3.56E-03
GO:0010638	Positive regulation of organelle organization	3.82E-03
GO:0005509	Calcium ion binding	4.00E-03
GO:0032535	Regulation of cellular component size	4.61E-03
GO:0015980	Energy derivation by oxidation of organic compounds	4.74E-03
GO:0009605	Response to external stimulus	4.94E-03
GO:0071229	Cellular response to acid chemical	5.25E-03
GO:0071396	Cellular response to lipid	6.09E-03
GO:0010026	Trichome differentiation	6.35E-03
GO:0009755	Hormone-mediated signaling pathway	7.01E-03
GO:0019752	Carboxylic acid metabolic process	7.24E-03
GO:1901564	Organonitrogen compound metabolic process	7.57E-03
GO:0005982	Starch metabolic process	7.75E-03
GO:0009630	Gravitropism	7.75E-03

Supplementary Table 16. Enriched GO terms in leaf-specific genes.

Gene Ontology ID	Gene Ontology term	Enrichment FDR
GO:0009535	Chloroplast thylakoid membrane	2.76E-22
GO:0009570	Chloroplast stroma	8.67E-15
GO:0006098	Pentose-phosphate shunt	5.00E-12
GO:0009941	Chloroplast envelope	8.78E-12
GO:0009523	Photosystem II	4.00E-11
GO:0016168	Chlorophyll binding	7.88E-11
GO:0018298	Protein-chromophore linkage	7.17E-10
GO:0003682	Chromatin binding	2.07E-09
GO:0009522	Photosystem I	2.33E-09
GO:0000785	Chromatin	2.68E-09
GO:0009765	Photosynthesis, light harvesting	1.93E-08
GO:0009902	Chloroplast relocation	3.62E-08
GO:0003677	DNA binding	8.47E-08
GO:0005634	Nucleus	1.07E-07
GO:0006364	rRNA processing	1.54E-07
GO:0010218	Response to far red light	3.08E-07
GO:0010207	Photosystem II assembly	3.66E-07
GO:0019288	Isopentenyl diphosphate biosynthetic process, Methylerythritol 4-phosphate pathway	6.03E-07
GO:0035304	Regulation of protein dephosphorylation	6.06E-07
GO:0010027	Thylakoid membrane organization	6.97E-07
GO:0048038	Quinone binding	7.24E-07
GO:0010228	Vegetative to reproductive phase transition of meristem	8.88E-07
GO:0009637	Response to blue light	2.88E-06
GO:0010114	Response to red light	4.02E-06
GO:0015976	Carbon utilization	4.02E-06
GO:0010319	Stromule	4.11E-06
GO:0015995	Chlorophyll biosynthetic process	8.93E-06
GO:0009773	Photosynthetic electron transport in photosystem I	1.38E-05
GO:0010155	Regulation of proton transport	2.90E-05
GO:0009862	Systemic acquired resistance, salicylic acid mediated signaling pathway	6.43E-05
GO:0005667	Transcription factor complex	8.43E-05
GO:0042744	Hydrogen peroxide catabolic process	9.49E-05
GO:0048046	Apoplast	1.20E-04
GO:0033554	Cellular response to stress	1.27E-04
GO:0016757	Transferase activity, transferring glycosyl groups	1.83E-04
GO:0015937	Coenzyme A biosynthetic process	2.77E-04
GO:0042793	Transcription from plastid promoter	2.78E-04
GO:0019344	Cysteine biosynthetic process	2.80E-04
GO:0015986	ATP synthesis coupled proton transport	2.89E-04
GO:0045893	Positive regulation of transcription, DNA-templated	3.07E-04
GO:0006417	Regulation of translation	3.11E-04
GO:0044272	Sulfur compound biosynthetic process	3.32E-04
GO:0016117	Carotenoid biosynthetic process	3.82E-04
GO:0003700	Transcription factor activity, sequence-specific DNA binding	4.19E-04

GO:0016021	Integral component of membrane	4.73E-04
GO:0009965	Leaf morphogenesis	5.67E-04
GO:0015078	Hydrogen ion transmembrane transporter activity	5.90E-04
GO:0010035	Response to inorganic substance	5.92E-04
GO:0006563	L-serine metabolic process	6.29E-04
GO:0009867	Jasmonic acid mediated signaling pathway	6.29E-04
GO:0008266	Poly(U) RNA binding	6.71E-04
GO:0030154	Cell differentiation	7.09E-04
GO:0006636	Unsaturated fatty acid biosynthetic process	1.02E-03
GO:0072330	Monocarboxylic acid biosynthetic process	1.02E-03
GO:0016226	Iron-sulfur cluster assembly	1.24E-03
GO:0035639	Purine ribonucleoside triphosphate binding	1.56E-03
GO:0019321	Pentose metabolic process	1.60E-03
GO:0045156	Electron transporter, transferring electrons within the cyclic electron transport pathway of photosynthesis activity	1.65E-03
GO:0031348	Negative regulation of defense response	1.74E-03
GO:0005739	Mitochondrion	1.81E-03
GO:0006566	Threonine metabolic process	1.82E-03
GO:0050136	NADH dehydrogenase (quinone) activity	2.33E-03
GO:0006546	Glycine catabolic process	2.35E-03
GO:0009743	Response to carbohydrate	2.38E-03
GO:0005509	Calcium ion binding	2.58E-03
GO:0048481	Ovule development	2.65E-03
GO:0010310	Regulation of hydrogen peroxide metabolic process	2.96E-03
GO:0046983	Protein dimerization activity	2.99E-03
GO:0010154	Fruit development	3.48E-03
GO:0010158	Abaxial cell fate specification	4.33E-03
GO:0021700	Developmental maturation	4.35E-03
GO:0006096	Glycolytic process	4.73E-03
GO:0006094	Gluconeogenesis	4.87E-03
GO:0009651	Response to salt stress	4.91E-03
GO:0009908	Flower development	4.99E-03
GO:0005840	Ribosome	5.38E-03
GO:0045259	Proton-transporting ATP synthase complex	5.72E-03
GO:0030001	Metal ion transport	6.66E-03
GO:0009084	Glutamine family amino acid biosynthetic process	6.84E-03
GO:0042773	ATP synthesis coupled electron transport	6.93E-03
GO:0016614	oxidoreductase activity, acting on CH-OH group of donors	6.93E-03
GO:0008135	Translation factor activity, RNA binding	7.62E-03
GO:0032403	Protein complex binding	8.04E-03
GO:0016874	Ligase activity	8.23E-03
GO:0005215	Transporter activity	8.28E-03
GO:0015977	Carbon fixation	8.53E-03
GO:0032549	Ribonucleoside binding	8.61E-03
GO:0010264	Myo-inositol hexakisphosphate biosynthetic process	8.64E-03
GO:0006099	Tricarboxylic acid cycle	9.02E-03
GO:1901617	Organic hydroxy compound biosynthetic process	9.71E-03

References

1. Lespinasse, D. *et al.* A saturated genetic linkage map of rubber tree (*Hevea* spp.) based on RFLP, AFLP, microsatellite, and isozyme markers. *Theor. Appl. Genet.* **100**, 127-138 (2000).
2. Ohmiya, H. *et al.* RECLU: a pipeline to discover reproducible transcriptional start sites and their alternative regulation using capped analysis of gene expression (CAGE). *BMC Genomics* **15**, 269 (2014).
3. Chow, K.-S., Ghazali, A.-K., Hoh, C.-C. & Mohd-Zainuddin, Z. RNA sequencing read depth requirement for optimal transcriptome coverage in *Hevea brasiliensis*. *BMC Res. Notes* **7**, 69 (2014).
4. Rahman, A.Y. *et al.* Draft genome sequence of the rubber tree *Hevea brasiliensis*. *BMC Genomics* **14**, 75 (2013).
5. Xia, Z. *et al.* RNA-Seq analysis and *de novo* transcriptome assembly of *Hevea brasiliensis*. *Plant Mol. Biol.* **77**, 299-308 (2011).
6. Li, D., Deng, Z., Qin, B., Liu, X. & Men, Z. *De novo* assembly and characterization of bark transcriptome using Illumina sequencing and development of EST-SSR markers in rubber tree (*Hevea brasiliensis* Muell. Arg.). *BMC Genomics* **13**, 192 (2012).
7. Triwitayakorn, K. *et al.* Transcriptome sequencing of *Hevea brasiliensis* for development of microsatellite markers and construction of a genetic linkage map. *DNA Research: An International Journal for Rapid Publication of Reports on Genes and Genomes* **18**, 471-482 (2011).
8. Schulz, M.H., Zerbino, D.R., Vingron, M. & Birney, E. Oases: robust *de novo* RNA-seq assembly across the dynamic range of expression levels. *Bioinformatics* **28**, 1086-1092 (2012).

9. Lassmann, T. TagDust2: a generic method to extract reads from sequencing data. *BMC Bioinformatics* **16**, 1-8 (2015).

10. Li, H. & Durbin, R. Fast and accurate short read alignment with Burrows–Wheeler transform. *Bioinformatics* **25**, 1754-1760 (2009).
